# Different functional alteration in attention‐deficit/hyperactivity disorder across developmental age groups: A meta‐analysis and an independent validation of resting‐state functional connectivity studies

**DOI:** 10.1111/cns.14032

**Published:** 2022-12-05

**Authors:** Ningning Liu, Qianrong Liu, Ziqi Yang, Jie Xu, Guanghui Fu, Yi Zhou, Haimei Li, Yufeng Wang, Lu Liu, Qiujin Qian

**Affiliations:** ^1^ Peking University Sixth Hospital, Institute of Mental Health Beijing China; ^2^ NHC Key Laboratory of Mental Health (Peking University), National Clinical Research Center for Mental Disorders (Peking University Sixth Hospital) Beijing China; ^3^ Shenzhen Key Laboratory of Affective and Social Neuroscience, Magnetic Resonance Imaging Center, Center for Brain Disorders and Cognitive Sciences, Shenzhen University Shenzhen China; ^4^ Department of Biomedical Sciences of Cells & Systems, Section Cognitive Neuroscience, University Medical Center Groningen, University of Groningen Groningen The Netherlands

**Keywords:** ADHD, ALE, development, functional connectivity, meta‐analysis, resting state

## Abstract

**Background:**

Attention‐deficit/hyperactivity disorder (ADHD) is a highly complex and heterogeneous disorder. Abnormal brain connectivity in ADHD might be influenced by developmental ages which might lead to the lacking of significant spatial convergence across studies. However, the developmental patterns and mechanisms of ADHD brain connectivity remain to be fully uncovered.

**Methods:**

In the present study, we searched PubMed, Scopus, Web of Science, and Embase for seed‐based whole‐brain resting‐state functional connectivity studies of ADHD published through October 12th, 2020. The seeds meeting inclusion criteria were categorized into the cortex group and subcortex group, as previous studies suggested that the cortex and subcortex have different temporal patterns of development. Activation likelihood estimation meta‐analysis was performed to investigate the abnormal connectivity in different age groups (all‐age group, younger: <12 years, older: ≥12 years). Moreover, significant convergence of reported foci was used as seeds for validation with our independent dataset.

**Results:**

As with previous studies, scarce results were found in the all‐age group. However, we found that the younger group consistently exhibited hyper‐connectivity between different parts of the cortex and left middle frontal gyrus, and hypo‐connectivity between different parts of the cortex and left putamen/pallidus/amygdala. Whereas, the older group (mainly for adults) showed hyper‐connectivity between the cortex and right precuneus/sub‐gyral/cingulate gyrus. Besides, the abnormal cortico‐cortical and cortico‐subcortical functional connectivity in children, and the abnormal cortico‐cortical functional connectivity in adults were verified in our independent dataset.

**Conclusion:**

Our study emphasizes the importance of developmental age effects on the study of brain networks in ADHD. Further, we proposed that cortico‐cortical and cortico‐subcortical connectivity might play an important role in the pathophysiology of children with ADHD, while abnormal cortico‐cortical connections were more important for adults with ADHD. This work provided a potential new insight to understand the neurodevelopmental mechanisms and possible clinical application of ADHD.

## Graphical abstract

Abnormal brain connectivity in ADHD might be influenced by developmental ages which might lead to the lacking of significant spatial convergence across studies. Cortico‐cortical and cortical‐subcortical connectivity might be an essential aspect in the pathophysiology of children with ADHD, while abnormal cortico‐cortical connections were more important for older ADHD (mainly for adults with ADHD).

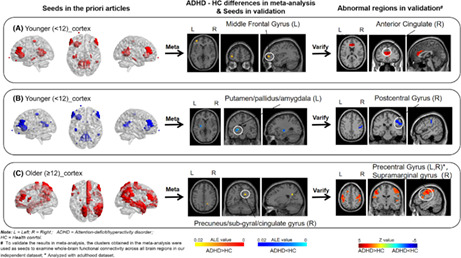

## 1 INTRODUCTION

Attention‐deficit/hyperactivity disorder (ADHD) is a complex and heterogeneous disorder in children/adults, which has a significant impact on the patients, their families, and society.^1^ Though recent developments in neuroimaging technology have advanced a deeper understanding of ADHD, the conclusions among these studies are not all consistent, and it is still difficult to translate scientific knowledge to high‐impact clinical applications.

### 1.1 Age contributes to the inconsistent findings of neuroimaging study in ADHD

Human brain development is a promising and challenging topic in neuroscience. During human growth and development, the brain undergoes a series of complex and coordinated processes such as neural differentiation, synaptogenesis, myelination, and synaptic pruning.^2^, ^3^ Meanwhile, the psychosocial changes are parallel to structural and functional changes of brain.^4^ Abnormal brain development may be related to several diseases such as autistic spectrum disorder (ASD),^5^, ^6^, ^7^ schizophrenia,^8^, ^9^ and ADHD.^10^, ^11^ A growing number of studies have suggested that development might be a good entry point to explore the possible mechanisms of ADHD, and knowing the developmental track of ADHD is crucial for effective diagnosis and treatment.

Resting‐state functional magnetic resonance imaging (rs‐fMRI) is a non‐invasively effective tool for assessing the development of the human brain.^12^ It measures the resting‐state intrinsic fluctuations in neural activity which is thought to consume about 60%–80% of the energy used by the brain.^13^, ^14^ Compared with task‐related functional MRI, this convenient imaging method reduces the potential confounder of different ages and different symptoms because no explicit cognitive task or demands is needed during scanning. So, it is more suitable for studies involving different ages and varying severity of disease.^15^ Abnormal brain connectivity or network dysfunction has been considered as a good tool to understand psychiatric disorders.^16^

Though large sample size longitudinal structural brain abnormalities across the development of ADHD have been found in many studies, and former studies have demonstrated the significant association between brain structure and brain function,^17^ almost all functional connectivity (FC) abnormality associated with ADHD is conventionally defined by the case‐control differences with small sample size. Besides, studies in ADHD have mainly focused on abnormalities of either ADHD child alone or ADHD adult alone, and their findings are often mixed. The studies that considered neurodevelopment of the whole‐brain connectivity architecture remain limited.

Though there are already three meta‐analyses about resting‐state functional connectivity (rs‐FC) of ADHD, these results were not always consistent with each other. Two of them focused on predefined networks and found abnormal inter‐network and intra‐network connectivity in ADHD.^18^, ^19^ The other one found no significant spatial convergence.^20^ However, we note that with Multilevel Kernel Density Analysis method, Sutcubasi et al. reported that when the analysis was restricted to children and adolescents (studies with adults were excluded), additional reduced connectivity between default mode network (DMN) and cognitive control network, between affective network (AN) and salience network were found.^18^ Gao et al. also revealed that the ADHD‐related brain functional alterations were more prominent when being limited to non‐adult studies.^19^ Though only negative results were reported by Cortese et al., they also proposed that different age range of participants might explain the mixed findings across meta‐analyses.^20^ Despite different study enrollments and approaches used in the existing meta‐analyses, their results consistently suggested a potential developmental effect on studies in ADHD.

### 1.2 Cortex and subcortex have different temporal patterns of development

Various brain networks and large‐scale intrinsic neural circuits including multiple cortico‐cortical and cortico‐subcortical regions have been found to be involved in ADHD.^21^ However, both human and animal studies have revealed cortex and subcortex might have different temporal patterns of development. FC tended to increase in cortical circuits during development, but subcortical circuits showed inconsistent results,^17^, ^22^, ^23^ and the development of cortical‐subcortical loop is complicated which may be varied in different regions.^24^, ^25^ Besides, the subcortex seems to be more conservative compared with cortex. Subcortex develops mainly during the early stage, especially children and adolescence, whereas cortex continues to develop after adolescence.^26^, ^27^

Recently, Casey et al. proposed hierarchical changes in the circuitry of the emotional brain: from subcortico‐subcortical in children, then to subcortico‐cortical/cortico‐subcortical in adolescence, and finally to cortico‐cortical in young adulthood.^28^ For ADHD, it has been argued that subcortical anomalies contribute to the onset of the disease and persist throughout life, while the variable clinical course of ADHD is dictated by the plasticity of the cerebral cortex.^29^, ^30^ All the studies suggested that brain development is spatiotemporally heterogeneous, and it may provide us additional information by focusing on subcortical and cortical circuit‐based changes with age.

Therefore, in this study, we aimed to make a meta‐analysis to explore the convergence of rs‐FC changes for ADHD from the perspective of development. Given the different development patterns of cortex and subcortex, here we divided the studies into two groups (cortex and subcortex) according to the localization of seeds. The main questions here are (1) whether could we find more spatially converge findings across studies after dividing the studies according to sample age and (2) how abnormal connections of subcortical and cortical circuits change with age. Finally, we also performed a validation experiment in our own dataset to verify the results in the meta‐analysis.

## 2 METHODS AND MATERIALS

### 2.1 Meta‐analysis

#### 2.1.1 Search strategy

Our literature search was conducted in PubMed, Scopus, Web of Science, and Embase databases on October 12, 2020 (including studies in‐press). Details of the search strategy were reported in the supplementary materials (Supplementary Appendix S1). In addition, we also checked for further information from the reference sections of the review article.

To explore the developmental effect, we defined “younger” as the mean age of individuals under 12 years old and “older” as 12 years old and older. The article was bounded by 12 years old for the following reasons. Firstly, according to the latest diagnostic criteria of ADHD in DSM‐5, inattentive or hyperactive‐impulsive symptoms should be present prior to age 12 years. Secondly, a previous study reported that the youngest and middle tertile (<12) showed the largest case‐control differences in surface area and cortical thickness, respectively.^31^ In addition, a recent study found that the principal functional connectivity gradient in adolescents aged above 12y revealed an adult‐like form.^32^

See Supplementary Appendix S1 and Table S1 for study eligibility criteria and data extraction.

#### 2.1.2 Statistical analysis

GingerALE v3.0.2 was used to investigate brain connectivity differences between ADHD and healthy control (HC). Activation likelihood estimation (ALE) is a widely used technique for coordinate‐based meta‐analyses. See Supplementary Appendix S1 for more description of the ALE.

Two principal analyses were conducted. Firstly, to detect spatial convergence of cortex and subcortex in patients with ADHD, we performed analyses in the following 4 groups: “Cortex_ADHD > HC (studies for seeds in the cortex and ADHD > HC, and so on),” “Cortex_ADHD < HC,” “Subcortex_ADHD > HC,” and “Subcortex_ADHD < HC.” Secondly, we divided these four groups into “younger” and “older” based on their mean age, respectively, and repeated the above analyses. As recommended by Veronika I. Müller, cluster‐level FWE (voxel‐level: *p* < 0.001; cluster‐level: *p* < 0.05) was set.^33^ In the present study, analyses were run when more than 10 experiments were available. Given our hypothesis of potential developmental effects, we made one exception where there were 8 experiments available with “cortex_older_ADHD > HC.” To identify whether the results are driven by experiments featuring a specific age range or brain region,^33^ we also reported the contributing seeds in our studies to provide a more specific interpretation of the results.

### 2.2 Validation study

To validate the results by the meta‐analysis, the clusters obtained in the meta‐analysis were used as seeds to examine whole‐brain FC across all brain regions in our independent dataset.

#### 2.2.1 Participants

60 children with ADHD and 89 HC, 63 adults with ADHD, and 65 HC were recruited. All participants were diagnosed by qualified psychiatrists at the Peking University Sixth Hospital. Due to poor scan quality, the data that finally entered the statistical analysis included 52 children with ADHD and 87 HC, and 62 adults with ADHD and 64 HC (Table 1). Part of the data has been used in our previous work.^34^ For more details about the participants and MRI analysis see supplementary materials (Supplementary Appendix S2). This study was approved by the Research Ethics Review Board of Peking University Sixth Hospital. Written informed consent was obtained from the parents of children and adult participants.

**TABLE 1:** Demographic characteristics of validation study

	Adults				Children			
	ADHD (mean ± SD)	HC (mean ± SD)	Statistic	*p*‐value	ADHD (mean ± SD)	HC (mean ± SD)	Statistic	*p*‐value
N (M/F)	62 (40/22)	64 (38/26)	0.353	0.552	52 (46/6)	87 (81/6)	0.889	0.346
Age (years)	25.81 ± 4.47	27.00 ± 4.52	−1.489	0.139	10.36 ± 2.55	10.22 ± 2.58	0.285	0.776
IQ score	120.55 ± 9.69	121.67 ± 8.82	−0.681	0.497	106.13 ± 15.18	103.34 ± 14.69	1.070	0.286
Head motion/mm	0.53 ± 0.23	0.48 ± 0.28	1.152	0.379	0.59 ± 0.26	0.53 ± 0.24	1.353	0.178

#### 2.2.2 Statistical analysis

The two‐sample t‐test in the RESTplus statistical analysis module is used to compare the FC statistical parameter graphs of adults and children in ADHD and HC groups. The Gaussian random field (GRF) correction was performed to correct for multiple statistical comparisons, voxel *p* < 0.005 and cluster *p* < 0.05 were set. Sex, age together with IQ were entered as covariates in between‐group analyses. For more details see Supplementary Appendix S2.

## 3 RESULTS

### 3.1 Meta‐analysis

**FIGURE 1:**
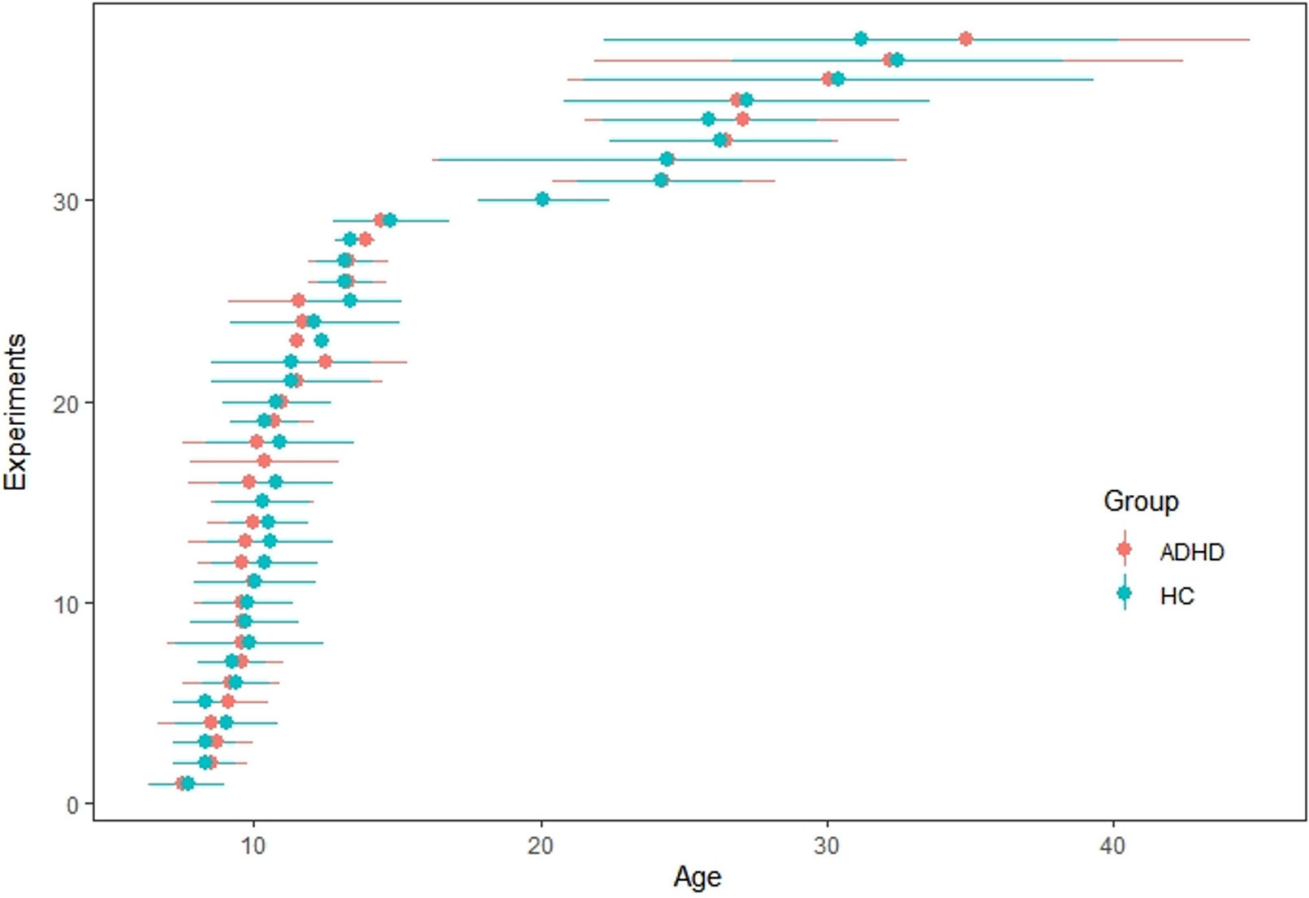
Mean age distribution of included study. Retained studies comprised 2407 participants: 1126 subjects with ADHD (age in years: 13.79 ± 7.99) and 1276 HCs (age in years: 15.05 ± 7.37). The age range of the populations in the included studies was 5–52 years old. As indicated in the figure, the available studies mainly focused on the population under 15 years old. Studies included in the age over 15 years old were rare, especially the range 15–20 years old.

Figure S1 in Supplementary Appendix S6 is the Preferred Reporting Items for Systematic Reviews and Meta‐Analyses (PRISMA) flow chart of the study. 35 studies (38 experiments) met the inclusion criteria. The demographic characteristics and methods of the studies included in the meta‐analysis are shown in Supplementary Appendix (Tables S2 and S3 in Supplementary Appendix S3). Figure 1 shows the age distribution of each included study.

#### 3.1.1 Results of Meta‐analysis

The anatomical location of foci reported in prior articles is shown in Supplementary material (Figures S2 and S3 in Supplementary Appendix S6). First, dividing the studies into cortex and subcortex only showed aberrant activity of left putamen/pallidus in the “Cortex_ADHD < HC” group (Figure S4 in Supplementary Appendix S6) (Table 2).

**TABLE 2:** Regions of abnormal brain connectivity between ADHD and HC in meta‐analysis (voxel‐level: *p* < 0.001; cluster‐level: *p* < 0.05)

Seeds anatomy	Subgroups	Effect anatomy	Effect network	Max.ALE	Local maximum^a^
					*X Y Z*
Fusiform gyrus, **Superior occipital lobe, Motor cortex (L), Superior temporal gyrus (L)**, Premotor cortex, FEF, right IPS, insula, TPJ, VFC, ACC, **Dorsal superior frontal gyrus (R), orbitofrontal cortex (L)**, Cingulate (L), DLPFC, PFC, Frontal operculum (R), FPN, MFG, **Ventral superior frontal gyrus (L)**, Precuneus, DMN, PCC.	Cortex_ADHD < HC	Putamen/pallidus (L)	AN	0.027	−30, −12, 2
**Dorsal superior frontal gyrus (R), Orbitofrontal cortex (L), DLPFC，Ventral superior frontal gyrus (L)**, Insula, TPJ, ACC, Frontal operculum (R); Supramarginal gyrus (R), Retrosplenial cortex, PCC	Cortex_younger_ADHD > HC	MFG (L)	FPN	0.018	−36, 52, 4
**Superior occipital lobe (L), Motor cortex, Superior temporal gyrus, Dorsal superior frontal gyrus (R), Orbitofrontal cortex (L), Ventral superior frontal gyrus (L)**, Insula, TPJ, aPFC, Frontal operculum (R), DLPFC, MFG, PCC	Cortex_younger_ADHD < HC	Putamen/pallidus/amygdala (L)	AN	0.027	−30, −12, 2
**FEF, ACC, DLPFC, Precuneus**, Superior temporal gyrus, insula, Middle temporal gyrus, PCC, Temporopolar area, Angular gyrus, IFC pars opercularis, RLP, MPFC	Cortex_older_ADHD > HC	Precuneus/sub‐gyral/cingulate gyrus (R)	DMN	0.013	18, −54, 32

*Note*: Bold font indicates contributing seeds, which is computed by the ratio of the summarized test values of all voxels of a specific cluster with and without the experiment in the analysis.

Abbreviations: ACC, anterior cingulate cortex; ADHD, attention‐deficit/hyperactivity disorder; AN, affective network; aPFC, anterior prefrontal cortex; DLPFC, dorsolateral prefrontal cortex; DMN, default mode network; FEF, Frontal eye field; FPN, frontoparietal network; HC, health control; IFC, inferior frontal cortex; IPS, intraparietal sulcus; L, left; MFG, middle frontal gyrus; MPFC, medial prefrontal cortex; NAcc, nucleus accumbens; PCC, posterior cingulate cortex; R, right; RLP, right inferior parietal lobe; TPJ, temporal parietal junction; VFC, ventral frontal cortex.

a Montreal Neurological Institute standard stereotaxic spaces.

The patients were further grouped based on mean age (<12 years and ≥12 years). The subsequent analyses were conducted only for “Cortex_younger_ADHD > HC,” “Cortex_older_ADHD > HC,” “Subcortex_younger_ADHD > HC,” “Cortex_younger_ADHD < HC,” and “Subcortex_younger_ADHD < HC,” as others could not provide at least 8 studies per group. Detailed information of ALE analyses could be found in the Supplementary materials (Table S4 in Supplementary Appendix S4).

**FIGURE 2:**
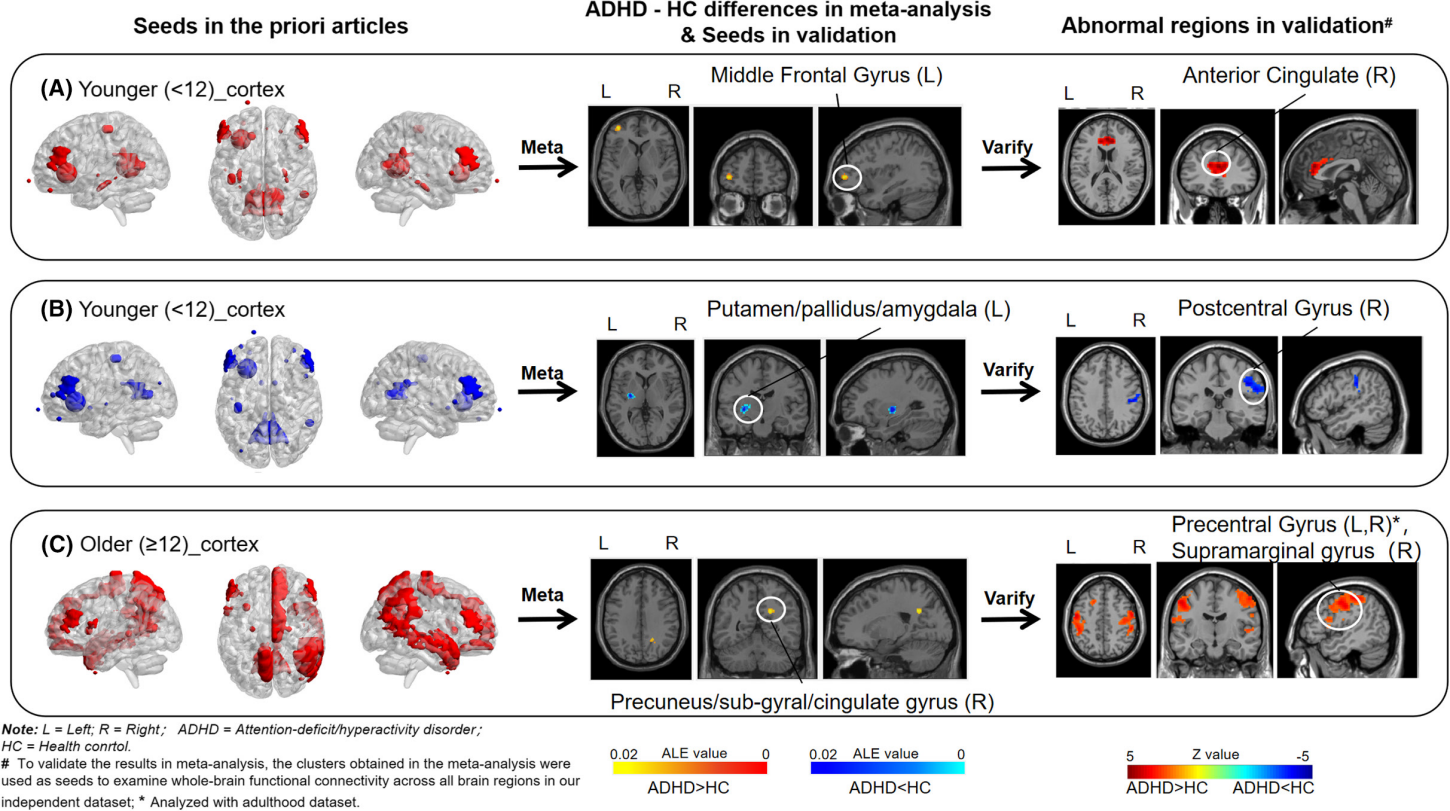
Brain regions of abnormal resting‐state functional connectivity in meta‐analysis and validation study in ADHD of two age groups. (A) In the meta‐analysis, ADHD group showed hyper‐connectivity between different seeds in the cortex and left middle frontal gyrus (MFG) (552 mm^3^ from (−44, 46, 0) to (−32, 56, 8) centered at (−37.5, 51.7, 3.3) with 1 peak, 89.9% middle frontal gyrus, 7.2% sub‐gyral, 1.4% inferior frontal gyrus, 1.4% superior frontal gyrus); In the validation study, ADHD shows increased FC between MFG and right anterior cingulate; (B) In the meta‐analysis, ADHD group showed hypo‐connectivity between seeds in the cortex and left putamen/pallidus/amygdala (Cluster 1: 1192 mm^3^ from (−34, −16, −4) to (−20, −6, 8) centered at (−28.7, −11.9, 1.8) with 2 peaks, 56.4% putamen, 14.8% lateral globus pallidus; Cluster 2: 544 mm^3^ from (−24, −10, −14) to (−16, −2, −6) centered at (−19.6, −6.6, −10.3) with 1 peak, 29.4% medial globus pallidus, 27.9% lateral globus pallidus, 5.9% amygdala); In the validation study, ADHD exhibited decreased FC between left putamen/pallidus/amygdala and right postcentral gyrus; (C). Meta‐analysis identified hyper‐connectivity between different seeds in the cortex and precuneus/sub‐gyral/cingulate gyrus (520 mm^3 from (16, −60, 28) to (26, −50, 34) centered at (20.3, −54.5, 31.4) with 2 peaks, 38.5% sub‐gyral, 33.8% precuneus, 27.7% cingulate gyrus) in the older group. In the validation study, adults with ADHD were characterized by increased FC between precuneus/sub‐gyral/cingulate gyrus and precentral gyrus, supramarginal gyrus.

For the younger group, we found that ADHD group showed hyper‐connectivity between different seeds in the cortex and left middle frontal gyrus (MFG) (Figure 2A), and hypo‐connectivity between seeds in the cortex and left putamen/pallidus/amygdala (Figure 2B). In the older group, ADHD was characterized by hyper‐connectivity between different seeds in the cortex and right precuneus/sub‐gyral/cingulate gyrus (Figure 2C) (Table 2).

Examining contributions of significant results indicated that for “Cortex_younger_ADHD > HC,” the results were mainly driven by studies with seeds in frontal cortex. For “Cortex_younger_ADHD < HC,” the abnormal connection was mainly driven by studies with seeds in temporoparietal cortex and frontal cortex. While for “Cortex_older_ADHD > HC,” the abnormal connectivity was driven by studies with seeds in frontoparietal lobe (Table 2). Besides, it should be noted that these contributing studies in the older group were all conducted in the adult populations.

### 3.2 Validation

For children, the FC from the left MFG as seed to the right anterior cingulate, which is in frontal cortex areas, was increased in the ADHD group when compared with that in the healthy matched individuals (Figure 2A). ADHD also showed decreased brain connectivity from left putamen/pallidus/amygdala to right postcentral gyrus, which is in the temporoparietal cortex (Figure 2B, Table 3).

**TABLE 3:** Regions of abnormal brain connectivity between ADHD and HC in validation study.

Seeds	Regions	Peak anatomical coordinate	Cluster size/voxels
		*X*, *Y*, *Z*	
**Younger (ADHD > HC)**			
Middle Frontal Gyrus (L)	Anterior Cingulate (R)	3, 36, 18	624
**Younger (ADHD < HC)**			
Putamen/pallidus/amygdala (L)	Postcentral Gyrus (R)	57, −21, 30	180
**Older (ADHD > HC)**			
Precuneus/sub‐gyral/cingulate gyrus (R)	Precentral Gyrus (R)	60, 0, 27	921
	Precentral Gyrus (L)	−51, −9, 45	1390
	Supramarginal Gyrus (R)	51, −48, 30	28

Abbreviations: ADHD, attention‐deficit/hyperactivity disorder; HC, health control; L, left; R, right.

For adults, patients with ADHD showed significant increased FC between right precuneus/sub‐gyral/cingulate gyrus and right precentral gyrus, left precentral gyrus, and right supramarginal gyrus, in the frontoparietal cortex (Figure 2C, Table 3).

That is to say, the results in the independent analysis not only validated the abnormal FC of cortex‐cortex and cortex‐subcortex in the meta‐analysis but also validated the corresponding brain areas in the meta‐analysis.

## 4 DISCUSSION

With ALE, we investigated the spatial convergence of ADHD‐related hyper‐ and/or hypo‐connectivities in the cortex and subcortex, across development (<12 and ≥12 years). In the all‐age group, we only found aberrant activity in the “Cortex_ADHD < HC” group. However, further analysis showed that for the younger group, the seeds in the cortex consistently showed hyper‐connectivity with middle frontal gyrus, whereas hypo‐connectivity with left putamen/pallidus/amygdala. While for the older group, more precisely, for adults, the cortex seeds showed a consistent hyper‐connectivity with right precuneus/sub‐gyral/cingulate gyrus. Moreover, the results of the independent analyses not only validated the abnormal cortex‐cortex and cortex‐subcortex FCs patterns observed in the meta‐analysis but also showed overlapped brain regions with that identified in the meta‐analyses.

As in the introduction, there are already three rs‐FC meta‐analyses about ADHD, which consistently suggested a potential developmental effect on FC in ADHD. However, no further effort was performed to elucidate the precise developmental influence in these studies. Compared with these studies, our current study enrolled more rs‐FC original studies (Tables S5 and S6 in Supplementary Appendix S5) and mainly focused on the developmental effects on the subcortical and cortical connectivity patterns in ADHD. Our findings supplemented the previous studies from a new point of view.

Our study showed that both the younger and the older patients with ADHD were with hyper‐connectivity among cortical regions, while the younger group also indicated additional hypo‐connectivity between cortex and subcortex. This might be explained by the existence of developmental delay in ADHD. Multiple analyses of FC showed that the development of large‐scale brain networks is presented with weakening of short‐range FC and strengthening of long‐range FC.^17^, ^35^ Increasing long‐range FC with age may be assisted by increased myelination, which continues into young adulthood.^36^ While the significant reduction in short‐range FC of regions in local networks may be partly related to selective synaptic pruning, which is traditionally known to occur from early infancy and well into the second decade of life.^37^, ^38^ Due to the delay in the maturation, ADHD would exhibit increased short‐range connections (within cortex) and decreased long‐range connections (between cortex and subcortex) compared with HC as found in our present study.

There was a substantial amount of structural‐developmental studies of brain with a large sample size in the last few decades. For cortex, Shaw et al. conducted a series of longitudinal brain imaging development research between ADHD and HC. They found that ADHD and HC have similar developmental trajectories, but the median age by which 50% of the cortical points attained peak thickness for the ADHD was 3 years later than HC,^39^ and the cortical area was about 2 years later than HC.^11^ Subsequently, coordinated analysis of large‐scale clinical and population‐based samples explored the development of surface area and thickness. Similarly, differences were only found for children.^31^ These studies all implied the existence of developmental delay for ADHD, especially in the frontal brain regions which were closely related to cognitive deficits in ADHD including response inhibition, attention, and higher‐order motor control.^40^, ^41^ Notably, in our meta‐analysis and validation analysis, abnormal cortical connections were also mainly located in frontal brain regions. This might indicate that maturing structural networks are an initial foundation for the functional connections.^42^

In this study, spatial convergence in cortico‐subcortical connectivity was only found in the younger group and the older group even did not have enough data to conduct a meta‐analysis. This might suggest that rare attention has been paid to cortico‐subcortical connectivity in adult. Of course, it is also possible that few relevant positive findings have been found in the adult. This was consistent with previous case‐control structural studies of ADHD which showed small to medium effect sizes of subcortex for adolescence and adult.^43^, ^44^ Specifically, Hoogman et al. made a mega‐analysis of 23 sites and found that the volumes of the subcortical structures were smaller in ADHD. However, effect sizes were highest for children under 15 years.^43^ A study based on the ADHD‐200 database also suggested that children with ADHD are related to delay in subcortical maturation.^45^ That is, these findings only obtained consistent results of developmental delay of subcortex in children ADHD.^46^ So insufficient data of cortico‐subcortical functional connectivity in adults with ADHD in our results were not surprising.

Further, previous studies have shown that this developmental change in connectivity is associated with changes in symptoms of ADHD. Cortico‐striatal‐thalamo‐cortical (CSTC) loops are consistently implicated in many mental diseases.^47^, ^48^, ^49^ Disturbances in any of the structures along CSTC pathways could produce abnormal control movement selection and initiation, reinforcement, reward processing, and habit formation,^50^, ^51^, ^52^, ^53^ which in turn leads to the behavioral symptoms of ADHD.^54^, ^55^ Though amygdala is not a part of the anatomically‐defined CSTC loop, it is one important brain correlate of temperament domains and plays a key role in emotion and motivation.^56^ Besides, amygdala‐cortical connectivity may also play an important role in many other behaviors.^57^, ^58^, ^59^ Our results show that disrupted FC between cortex and putamen/pallidus/amygdala only exists in younger ADHD, rather than in adults. This was to some extent consistent with the clinical observation that many of the typical behaviors of children with ADHD declined as age increased.^60^

### 4.1 Limitations and future directions

In the present study, based on our assumption, we conducted a meta‐analysis from a new perspective of neurodevelopment. Besides, the results were well validated by our big dataset. Nevertheless, our results should be interpreted in light of some limitations.

Firstly, heterogeneity needs to be taken into account when interpreting our results. ADHD is a highly heterogeneous disease. An increasing number of studies have indicated individual differences in brain development.^61^, ^62^, ^63^, ^64^ Recently, a voxel‐based morphometry study of adults with ADHD found the overlap of more than 2% between the individuals with persistent ADHD was only observed in a few brain loci. This might suggest that previous small group differences in the adult ADHD might be explained by the biologically heterogeneous on the individual level, as the “average ADHD patient” does not sufficiently reflect the degree of inter‐individual variation that characterizes ADHD.^65^ Some^20^ argued that the lack of significant spatial convergence might be due to heterogeneity in study participants, experimental procedures, analytic flexibility, and ADHD pathophysiology. Our study may face the same issue.

Secondly, as with other meta‐analyses, we did not have sufficient numbers of original studies for more subgroup analysis in this study. At least 17–20 experiments were recommended to have sufficient power in ALE meta‐analyses,^33^, ^66^, ^67^ while a number of 10–15 experiments would allow to perform a “meaningful ALE analysis”.^68^ And, the required number of experiments is mainly dependent on the expected effect size. So in the present study, analyses were run when more than 10 experiments were available to guarantee a meaningful ALE analysis. However, due to the limited number of documents, we could not obtain more information of development, considering that more detailed subgroups by age could have a power problem. For example, adolescence is a period of great plasticity or flexibility in neural and cognitive function, which is associated with a vulnerability to psychopathology.^69^, ^70^, ^71^ Brain undergoes an important developmental process during adolescence, including prefrontal synaptic pruning, and higher‐order cognition‐related white matter tracts myelination.^72^, ^73^ Yet less work has focused on the FC alternation of adolescent with ADHD. So adolescence was not analyzed separately. We tried to address this limitation by examining the contributions of the significant results and we found that these contributing studies in the older group were all conducted in adult populations, which verified our conclusion to some extent. Still, our results may not represent all abnormal connectivity between ADHD and HC because of insufficient numbers of original studies. For example, previous studies have reported the potential modulatory effect of stimulants and atomoxetine on several intrinsic brain activity metrics.^74^ Due to the limited original articles, the analyses limited to studies including only non‐medication naïve patients is not feasible. However, the children and adults in our validation analyses were all medicine naïve, and this might suggest that medicine use would not significantly affect our conclusions. In addition, we would like to stress that more original studies are needed to get more powerful results and to address the above‐mentioned question and other important questions, including comorbidities and gender, which also contribute to the heterogeneity of ADHD as we mentioned above.

Finally, our analysis was based on cross‐sectional studies. It has been known that part of childhood patients would remit in adulthood. To wit, children with ADHD consist of both persistent ADHD and remit ADHD in adulthood.^31^ Many studies have found difference in genetic background between ADHD remitters and persisters.^75^, ^76^ Some scholars hold that different adolescent neural trajectories between remitters and persisters originate in childhood. These typical or anomalous neural features “carry forward” during adolescence and eventually lead to improvement even eventual remission or persistence of symptoms in ADHD.^30^ In our study, all the adult patients should be taken as persisted ADHD based on the current diagnostic criteria, so the observed age‐related differences may just be the traits of these specific subgroups. So to explore the development of brain function with ADHD, longitudinal cohort studies with a large sample and wide age range are needed in the future.

## 5 CONCLUSION

The present work assessed the developmental changes of aberrant brain connectivity in ADHD. We found more consistent activation when the studies were grouped by patients' age. Specifically, there were obvious differences between cortex and subcortex, and between younger (<12 years) and older (≥12 years). Besides, the abnormal cortico‐cortical and cortico‐subcortical functional connectivity in children and abnormal cortico‐cortical functional connectivity in adults were verified in our independent database. Our results emphasize the importance of age effect when studying the brain function of ADHD. We proposed that cortico‐cortical and cortical‐subcortical connectivity might be an essential aspect in the pathophysiology of children with ADHD, while abnormal cortico‐cortical connections were more important for older ADHD (mainly for adults with ADHD). This work allows us to better understand neurodevelopmental mechanisms of ADHD, which hold important implications for future prediagnosis and clinical treatment.

## FUNDING INFORMATION

This work was supported by the National Science Foundation of China (81571340, 81873802), the Capital's Funds for Health Improvement and Research (CFH: 2022‐2‐4144, 2020‐2‐4112), and the National Key Basic Research Program of China (973 program 2014CB846104).

## CONFLICT OF INTEREST

The named authors have no conflict of interest, financial or otherwise.

## Supporting information


AppendixS1

## Data Availability

The information of the original articles used in the study is included in the article/Supplementary Material; further inquiries can be directed to the corresponding author.
