# Supplementary material for: Different functional alteration in attention‐deficit/hyperactivity disorder across developmental age groups: A meta‐analysis and an independent validation of resting‐state functional connectivity studies
Source: CNS Neurosci Ther. 2022 Dec 5;29(1):60–9. doi: 10.1111/cns.14032 (PMC9804052; doi:10.1111/cns.14032)
Supplement: Supplementary file 1 — AppendixS1 [file CNS-29-60-s001.docx]

**Supplementary Material**

**Catalog**

[Appendix 1. Supplementary material for the methods of meta-analysis 2](#_Toc24818)

[Appendix 2. Supplementary material for the methods of validation study 5](#_Toc22083)

[Appendix 3. Summary of the studies included in meta-analysis 7](#_Toc6932)

[Appendix 4. Detail of ALE analyses 10](#_Toc4609)

[Appendix 5. Comparisons of the current results compatible with the previous meta-analysis 10](#_Toc2265)

[Appendix 6. Supplementary figures 16](#_Toc4171)

# Appendix 1. Supplementary material for the methods of meta-analysis

**Search strategy/syntax and results (hits) from each electronic database**

*Last search:* October *12^th^, 2020*

Pubmed:

- Search terms:

((resting[Title/Abstract]) OR (rest[Title/Abstract])) AND ADHD[MeSH Terms]

Limits: none

Results: 512 hits

Scopus:

- Search terms:

( ( TITLE-ABS-KEY ( adhd ) OR TITLE-ABS-KEY ( attention AND deficit AND disorder AND with AND hyperactivity ) ) ) AND ( ( TITLE-ABS-KEY ( rest ) OR TITLE-ABS-KEY ( resting ) ) )

Limits: none

Results: 987 hits

Web of Science:

- Search terms:

(TS=("ADHD") OR TS=("attention deficit/hyperactivity disorder")OR TS=("Inattention")OR TS=("hyperactivity") OR TS=("attention deficit")) AND TS=("rest*") AND TS=("connect*")

Timespan: All years. Databases: WOS, BIOSIS, CSCD, DRCI, DIIDW, INSPEC, KJD, MEDLINE, RSCI, SCIELO. Search language=Auto

Embace_381

- Search terms:

'attention deficit disorder'/exp AND 'functional connectivity'/exp

Limits: none

Results: 381 hits

**After merging and de-duplication: 1995 references**

**Study Eligibility Criteria**

**Inclusion criteria**

We included original fMRI studies if they (1) had a current formal diagnosis of ADHD according to the DSM-III, DSM-IV, DSM-IV-TR, DSM-5 or ICD-10 criteria; (2) contained a between-group “ADHD versus health control” contrast; and/or (3) correlate with individual level of ADHD symptoms (measured by Adult ADHD Self-Report Scale; Parental Account of Children's Symptoms; Conners' rating scales, and so on). When no peak coordinates of between-group effect were provided, we contacted the authors for detailed information.

**Exclusion criteria**

We excluded the studies if they were (1) not human studies; (2) not ADHD studies; (3) not fMRI studies; (4) not resting-state FC (rsFC) studies; (5) not written in English or Chinese; (6) dataset-based studies. Studies were also excluded if they (1) had no health controls; (2) were not seed-based whole-brain FC studies; (3) did not compare FC of ADHD with healthy control (HC); (4) the same sample and seed regions had been reported in another included study; (5) did not survive statistical correction; (6) had irretrievable peak coordinates when requested. Detail of exclusion studies were listed in the Tabel ***(Supplementary*** ***Appendix 1. Table)***.

**Data extraction**

First, the coordinates of each seed and the peak coordinates of regions exhibiting significant differences between the group were extracted. Then, for the coordinates reported in Talairach system, MNI space were obtained by GingerALE 3.0.2 (http://www.brainmap.org/ale/). Second, according to previous studies, all the results were divided into “hyper-connectivity (ADHD>HC)” and “hypo-connectivity (ADHD<HC)”[1]. Hyper-connectivity was defined as 1) increased positive or decreased negative rsFC in the ADHD group or 2) positive correlations between rsFC and levels of ADHD symptoms. Hypo-connectivity was defined as 1) decreased positive or increased negative rsFC in the ADHD group or 2) negative correlations between rsFC and levels of ADHD symptoms.

Then, the seeds meeting inclusion criteria (35 studies, 38 experiments) were categorized into 2 groups (cortex and subcortex) based on their locations. To explore the developmental effect, we defined “younger” as the mean age of individuals under 12 years old and “older” as 12 years old and older. The article was bounded by 12 years old for the following reasons: Firstly, according to the latest diagnostic criteria of ADHD in DSM-5, inattentive or hyperactive-impulsive symptoms should be present prior to age 12 years. Secondly, previous study reported that the youngest and middle tertile (<12) showed the largest case-control differences in surface area and cortical thickness, respectively[2]. In addition, a recent study found that the principal functional connectivity gradient in adolescents aged above 12y revealed adult-like form[3].

**GingerALE procedure**

Activation likelihood estimation (ALE) is a widely used technique for coordinate‐based meta‐analyses which is able to help us synthesize large amounts of neuroimaging data objectively. Based on modeling foci as probability distributions centered at the coordinates, ALE assesses the spatial convergence between focis[4]. In addition, it can estimate the contributions of each included experiment. Examining contributions can help to identify whether the results are driven by experiments featuring a specific age range or brain region, which would allow a more appropriate explanation of the results[5].

**Table S1. List of studies excluded after full-text screening, with reasons for exclusion.**

| Year | Author | Reason for Exclusion |
| --- | --- | --- |
| 2018 | Zhou et al. | ReHo,DC,VMHC |
| 2017 | Sudre et,al | ICA |
| 2014 | Jiang et,al | VMHC |
| 2013 | Yang et al. | ALFF，ReHo |
| 2010 | Fair et al. | ROI:ROI |
| 2007 | Cao et al. | ReHo |
| 2020 | Chen et.al | ROI:ROI |
| 2020 | Liu et al. | The same data and seed with "Integrity of Amygdala Subregion-Based Functional Networks and Emotional Lability in Drug-Naïve Boys With ADHD" |
| 2020 | Luo et,al | ROI:ROI |
| 2019 | Li et al. | Graph theoretical |
| 2019 | Rohr, C. S., | Cortico-cerebellar correlation |
| 2019 | Rubia et.al | No HC |
| 2018 | Cai et.al | Time-Varying |
| 2018 | de Lacy Nina et.al | Time-varying connectivity and whole-brain FNC using ICA in ADHD, |
| 2017 | Lee et.al | RI:ROI |
| 2016 | Wang et.al | ALFF |
| 2020 | Guo et.al | ROI:ROI |
| 2020 | Jeong et.al | ROI:ROI |
| 2020 | Jiang et,al | ALFF |
| 2020 | Shinya et.al | ROI matrix |
| 2020 | Picon et.al | No HC |
| 2020 | Ma, K., J. et.al | No HC |
| 2020 | Gustavo et.al | ROI matrix |
| 2020 | Phoebe et.al | Calculate Head Motion,no FC |
| 2020 | Michaela et.al | ROI:ROI |
| 2019 | Icer et.al | ICA |
| 2019 | Jiang et.al | DC and VMHC |
| 2019 | Jin et.al | mALFF |
| 2019 | Zepf et.al | ROIs matrix |
| 2019 | Henry et.al | Group Iterative Multiple Model Estimation |
| 2019 | Peter et.al | ICA |
| 2019 | Qian et.al | ICA |
| 2019 | Wang et.al | Clustering coefficient and shortest path length |
| 2019 | Yoo et.al | ICA |
| 2019 | Wang et.al | Global efficiency |
| 2019 | Wu et.al | ICA |
| 2019 | Dajani et.al | ICA |
| 2019 | Hearne et.al | ROIs matrix |
| 2019 | [Hongliang et.al](https://www.researchgate.net/scientific-contributions/2154205797-Hongliang-Zou) | Temporal variability |
| 2018 | Rosch et.al | ICA |
| 2018 | Qian et.al | No HC |
| 2018 | Shang et.al | ReHo |
| 2018 | Wang et.al | No HC |
| 2018 | Qian et.al | No HC |
| 2018 | Qian et.al | No HC |
| 2018 | Yoo et.al | ALFF |
| 2018 | Yoo et.al | ROIs matrix |
| 2018 | Kim et.al | ReHo |
| 2018 | Yang et.al | Unable to retrive coordinates |
| 2017 | Uytun et.al | Did not survive statistical correct |
| 2017 | Hasler et.al | ROIs matrix within DMN |
| 2017 | Tao et.al | ROIs matrix |
| 2017 | Silk et.al | ROIs matrix |
| 2017 | Cary et.al | Node Dissociation Index (NDI). |
| 2017 | Bos et.al | ICA |
| 2017 | Zhan et.al | ROIs matrix |
| 2017 | Sanefuji et.al | The study used ICA instead of SCA |
| 2017 | Akdeniz et.al | ICA |
| 2016 | Shang et.al | No HC |
| 2016 | Biskup et.al | ICA |
| 2016 | Yang et al. | No HC |
| 2016 | Sidlauskaite et.al | Network-based approach |
| 2016 | Yu et.al | ReHo |
| 2016 | Solai et.al | ICA |
| 2016 | Benli et.al | Unable to retrive coordinates |
| 2016 | Wang et.al | Graph-theory， regional functional connectivity strength |
| 2015 | Francx et.al | Within executive control network |
| 2015 | Ho et.al | ICA |
| 2015 | Krishna et.al | No comparation of FC between ADHD and HC |
| 2015 | Kim et.al | No HC |
| 2015 | Barber et.al | Mean value of severy seeds |
| 2014 | Alonso et.al | ICA |
| 2014 | Dey et.al | SNM |
| 2014 | Gates et.al | Group Iterative Multiple Model Estimation (GIMME） |
| 2014 | Elseline et.al | Did not survive statistical correct |
| 2014 | Shekarchi et.al | ICA |
| 2014 | Aaron et.al | Unable to retrive coordinates |
| 2014 | Ou et.al | About a novel computational framework |
| 2013 | Sokunbi et.al | Entropy |
| 2013 | Di et.al | DC,EC |
| 2013 | Li et.al | ReHo and ALFF |
| 2013 | Li et.al | ReHo |
| 2013 | Lori et.al | No HC |
| 2012 | Cocchi et.al | ROIs matrix |
| 2012 | Sato et.al | Unsupervised machine learning using a one-class support vector machine (OC-SVM) |
| 2012 | Chabernaud et.al | Unable to retrive coordinates |
| 2011 | Qiu et.al | ICA |
| 2011 | Yang et.al | ALFF |
| 2010 | Fair et al. | ROI:ROI |
| 2009 | Wang et.al | Topological architectures |
| 2008 | Tian et.al | RSAI |
| 2008 | Uddin et.al | Network homogeneity |
| 2008 | Zhu et.al | Discriminative analysis，reho |
| 2007 | Zang et.al | ALFF |
| 2006 | Cao et.al | ReHo |
| 2020 | Brennan et.al | No HC |
| 2019 | Chen et.al | graph theory |
| 2013 | Choi et.al | ICA |
| 2019 | Luke et.al | structure-function decoupling |
| 2020 | Robert et.al | correlated the connectivity to PRS（polygenic risk score） |
| 2014 | Jiang et.al | DC |
| 2012 | Kelly et.al | No HC |
| 2018 | Lin et.al | ROIs matrix |
| 2016 | Kevin et.al | No comparation of ADHD and HC，only compared left SM1 with right SM1 |
| 2019 | Pan et.al | Adaptive Sparse Representation (ASR) method and graph theory |
| 2019 | Pretus et.al | SFC（Stepwise Functional Connectivity) |
| 2017 | Rohr et.al | not whole brain |
| 2018 | Saletin et.al | Brain signal variability, |
| 2017 | Son et.al | ROIs matrix |
| 2020 | Sun et.al | ROI：ROI |
| 2019 | Tan et.al | Functional connectivity strength |
| 2019 | Wang et.al | Graph-theory， regional functional connectivity strength |
| 2018 | Yoo et.al | ALFF |
| 2016 | [Jae et.al](https://www.researchgate.net/scientific-contributions/2110884781-Jae-Hyun-Yoo) | ICA |
| 2013 | Yu et.al | Phase synchrony degree |
| 2008 | Zou et.al | ALFF |

# Appendix 2. Supplementary material for the methods of validation study

**Participants**

**Children:** For the sample of children, 60 children with ADHD and 89 HCs aged 6-16 were recruited. All children were diagnosed by a qualified child and adolescent psychiatrist at the Peking University Sixth Hospital’s Child and Adolescent Psychiatric Clinic from Child and Adolescent Psychiatric clinics of Peking University Sixth Hospital. The clinical diagnosis of ADHD and comorbidities were evaluated using the Diagnostic and Statistical Manual of Mental Disorders, 4th Edition (DSM-IV)[5]. ADHD rating scale-IV (ADHD RS-IV) was used to measure the inattention and hyperactivity/impulsivity symptoms. All subjects were with a full-scale IQ above 80 assessed using the Chinese Wechsler Intelligence Scale for Children (CWISC) [7] . All children have never been treated with drugs for ADHD and have never used other psychiatric drugs. The exclusion criteria were as follows: (1) Past or current childhood schizophrenia, affective disorder, autism, mental retardation, or epilepsy; (2) Severe physical, neurological abnormalities, consciousness disorders, or current substance abuse or dependence. In addition, due to excessive head movement (a translation greater than 3 mm in any direction or a rotation angle greater than 3 degrees), eight ADHD participants were excluded from further analysis. Healthy children were recruited from the local primary school as healthy controls (HCs). Due to poor scan quality, two healthy control was excluded. The data that finally entered the statistical analysis included 52 children with ADHD and 87 HCs.

**Adult:** For the sample of adults, 63 adults with ADHD and 65 HCs were recruited. All patients were diagnosed by qualified psychiatrists in the outpatient clinic of Peking University Sixth Hospital. Using the Structured Clinical Interview for DSM‐IV Axis I Disorders (SCID‐I) screening for any potential comorbidities. Conner's Adult ADHD Diagnostic Interview for DSM‐IV was used to confirm the diagnosis of ADHD. In addition, to assess the severity of ADHD symptoms, participants also completed the ADHD Rating Scale‐IV (ADHD RS-IV). All subjects were with a full-scale IQ above 90 assessed using the Wechsler Adult Intelligence Revised in China (WAISRC). All adults have never been treated with drugs for ADHD and have never used other psychiatric drugs. The exclusion criteria were as follows: (1) Past or current suffering from schizophrenia, affective disorder, autism, mental retardation, or epilepsy; (2) Severe physical, nervous system abnormalities, disturbance of consciousness, and current substance abuse or dependence. Due to excessive head movement, the data of one patient and one control were excluded from further analysis. The adult data that finally entered the statistical analysis included 62 adults with ADHD and 64 HCs.

**Data Acquisition**

MRI data of children were acquired using a Siemens Trio 3T scanner (Siemens, Erlangen, Germany) at the Imaging Center for Brain Research, Beijing Normal University. Functional images were acquired using an echo-planar imaging sequence with the following parameters: repetition time (TR) = 2000 ms, echo time (TE) = 30 ms, flip angle(FA) = 90°, thickness/skip = 3.5/0.7 mm, matrix = 64 × 64, field of view (FOV) = 200 × 200 mm, 33 axial slices and 240 volumes. High-resolution T1-weighted anatomical images were acquired with the following parameters: TR = 2,530 ms, TE = 3.39 ms, inversion time(TI) = 1,100 ms, FA = 7°, 128 slices, slice thickness = 1.33 mm, FOV = 256 × 256 mm, matrix = 256 × 256.

MRI data of adults were acquired using a 3T MR system (General Electric; Discovery MR750) in the Center for Neuroimaging in Peking University Sixth Hospital. Functional images were acquired using a gradient‐echo single‐shot echo planar imaging (GRE‐SS‐EPI) sequence with the following parameters: TR = 2000 ms, TE=30 ms, FA = 90°, slice thickness = 3.2 mm with no gap; matrix = 64 × 64; FOV = 220 × 220 mm; 43 axial slices and 240 volumes. High‐resolution T1‐weighted anatomical images were acquired with the following parameters: TR= 6.66 ms; TE = 2.93 ms; TI = 450 ms; FA= 8°; FOV = 256 × 256 mm; matrix = 256 × 256; slice thickness = 1.0 mm with no gap, 180 sagittal slices. During scanning, participants were asked to lay down with their eyes closed, think of nothing but without falling asleep.

**Data Preprocessing**

The data analysis was performed using the RESTplus [8] on MATLAB R2014a platform. The data preprocessing including following steps: (1) excluding the first 10 time points, (2) slice timing, (3) head motion correction, (4) spatial normalization: co-registration of individual T1 images to functional images, spatial normalization to the Montreal Neurological Institute (MNI) space (resampled voxel size = 3 × 3 × 3 mm3), (5) spatial smoothing (Gaussian kernel full-width at half-maximum = 6 mm), (6) linear detrending, (7) nuisance covariates regression: including 24 head motion parameters and global mean signals, and (8) filtering (0.01-0.08 Hz). This validation analysis was performed separately for children and adults, without all age-groups pooled together for analyses. Data harmonization was not applicable for the present study and was not performed in the analyses.

**Statistical analysis**

To validate the results in meta-analysis, the clusters obtained in the meta-analysis were used as seeds to examine whole-brain functional connectivity across all brain regions in our independent dataset. The FC analysis was conducted using RESTplus. The anatomical automatic labeling (AAL) template in the RESTplus software package was used to extract the average time series of the ROI, the Pearson’s correlation coefficients was calculated between the mean time series of the ROI and the time series of each voxel in the whole brain. Then the Fisher z transformation was used to improve the normality of the correlation coefficient.

The two-sample t test in the RESTplus statistical analysis module is used to compare the FC statistical parameter graphs of adults and children in ADHD and HC groups. The built-in brain mask of RESTplus (61 × 73 × 61 mm) was used as the registration template, and the initial threshold was *P* <0.05. The Gaussian random field (GRF) correction was performed to correct for multiple statistical comparisons, voxel *P* < 0.005 and cluster *P* < 0.05 were set. Sex, age together with IQ were entered as covariates in between-group analyses.

# Appendix 3. Summary of the studies included in meta-analysis

**Table S2. Summary of demographic characteristics of studies included in meta-analysis**

| Author | Nation | Year | Medicine | Hand | Comorbidities | IQ（ADHD/HC） | Age | | Sample sizes | | Sex | | Subtypes |
| --- | --- | --- | --- | --- | --- | --- | --- | --- | --- | --- | --- | --- | --- |
|  |  |  |  |  |  |  | ADHD | HC | ADHD | HC | ADHD | HC |  |
| Arfuso et al. | USA | 2019 | Abstained from taking them for 72 h prior to the initial assessment and scan. | */* | 28ODD，19Any anxiety disorder，11Any depressive disorder/5 Any anxiety disorder | 104.5±16.4/110.4±11.8 | 7.5±1.2 | 7.7±1.3 | 75 | 37 | 59：16 | 30：7 | 53C：10I：13H |
| Karalunas et al. | Portland. | 2014 | 37% taking stimulant | / | / | 106.7±13.2/114.1±13.2 | 8.48±1.26 | 8.3±1.1 | 11 | 15 | / | / | / |
| Karalunas et al. | Portland. | 2014 | 37% taking stimulant | / | / | 107.1±14.7/114.1±13.2 | 8.73±1.23 | 8.3±1.1 | 10 | 15 | / | / | / |
| Zhou et al. | China | 2019 | Naive | right | none | 115.49±15.78/121.19±11.61 | 8.51 ± 1.86 | 9.05 ± 1.80 | 47 | 21 | boy | boy | / |
| Karalunas et al. | Portland. | 2014 | 37% taking stimulant | / | / | 111.6±14.4/114.1±13.2 | 9.1±1.38 | 8.3±1.1 | 18 | 15 | / | / | / |
| Yang et al. | China | 2013 | Naive | right | none | ≥80 | 9.2±1.7 | 9.4±1.2 | 30 | 30 | / | / | C:I:H=30:0:0 |
| Dias et al. | Portland | 2013 | Washout of five half-lives | right | / | 109.24±14.93/116.91±12.82 | 9.57±1.47 | 9.21±1.2 | 35 | 64 | 27：8 | 39：25 | all C |
| Hong et al. | Australia | 2015 | 73 naive | 74right(ADHD)/20right(HC) | Oppositional defiant :16;Anxiety disorder:2/0 | 106.54±13.56/114.64±10.54 | 9.58±2.61 | 9.84 ± 2.57 | 83 | 22 | 65：18 | 14：8 | 44C,32I,1HI,not otherwise specified:6 |
| Kumar et al. | India | 2020 | Naive | right | / | 92.8±2.9/109.3±4.1 | 9.6 ±1.8 | 9.7 ±1.9 | 16 | 16 | male | male | / |
| Jiang et al. | China | 2018 | Naive | right | none | ≥80 | 9.6±1.7 | 9.8±1.6 | 30 | 33 | 18：12 | 18:15 | / |
| Lin et al. | Taiwan | 2015 | Stop took methylphenidate for at least one week before and during all assessments | right | 8ODD | 109.88±9.01/113.64±9.05， | 9.94±1.77 | 10.04±2.13 | 25 | 25 | 20：5 | 19：6 | / |
| Wang et al. | China | 2015 | / | right | none | 102.48±15.76/112.78±11.69 | 9.6 ±1.57 | 10.36±1.87 | 30 | 33 | / | / | C:I:H=30:0:0 |
| Mizuno et al. | Japan | 2017 | 13 were naive. All participants were medication-free prior to MRI for at least 5 times half-lives | 26：5/28：2 | 3ODD | 97.4±14.8/103.8±10.2 | 9.7±2.0 | 10.6±2.2 | 31 | 30 | male | male | C:I:H=24:7:0 |
| Posner et al. | USA | 2014 | Naive | / | 6 ODD; 1 SAD/- | 95.8±17.7/99.9±19.0 | 10.0±1.6 | 10.5±1.4 | 22 | 20 | 17：5 | 15：5 | C:I:H=19:3:0 |
| Yu et al. | China | 2016 | Naive | right | 11ODD | 106 ± 16/121 ± 14 | 10.31 ± 1.79 | 10.30 ± 1.68 | 35 | 30 | male | male | C:I:H=16:18:1 |
| Posner et al. | USA | 2014 | Naive | / | 4ODD/CD2ODD/CD;MDD,1ODD/CD,SAD;1ODD/CD,enuresis;1ODD/CD,MDD,GAD;1MDD;1 enuresis | 99.12±15.82 /109.10±15.74 * | 9.83±2.12 | 10.77±1.98 | 30 | 31 | 24：6 | 21：10 | 24C:5I |
| Xie et al. | china | 2016 | / | matched | None | ≥80 | 10.4±2.6 | / | 10 | 10 | boy | boy | / |
| Li et al. | China | 2014 | Naive | right | None | <= 90 | 10.1±2.6 | 10.9±2.6 | 33 | 32 | male | male | C:I:H=22:11:0 |
| Kim et al. | Korea | 2017 | Medication-naïve or drug-free for 6 months prior to the study, | / | None | 96.0±9.7/96.2±8.3 | 10.7±1.4 | 10.4±1.2 | 13 | 13 | 10:03 | 9:04 | C:I:H=3:4:6 |
| Mennes et al. | USA | 2011 | / | / | Four with ADHD had comorbid oppositional deﬁant disorder, and one had comorbid adjustment disorder with depressive mood. | 111.8±14.26/112.1±14.11 | 11.0±1.26 | 10.8±1.92 | 17 | 17 | 14：3 | 9：8 | C:I:H=11:6:0 |
| McLeod et al. | Canada | 2015 | Refrain from taking their medication on the day of assessment. No children in the control and DCD groups were on stimulant medication, 11 of 21 the children in the ADHD group were on stimulant medication。 | 2 left/4 left（ADHD+DCD) | None | 105.4±11.8/113.0±13.4/104.8 ± 15.8(ADHD+DCD) | 11.5±3.0 | 11.3±2.8 | 18 | 23 | 14：4 | 11：12 | / |
| McLeod et al. | Canada | 2015 | Refrain from taking their medication on the day of assessment. No children in the control and DCD groups were on stimulant medication, 9 of 18 children in the DCD ADHD group were on stimulant medication. | 2 left/2 left | None | 105.4±11.8/113.0±13.4/104.8 ± 15.8(ADHD+DCD) | 12.5±2.9 | 11.3±2.8 | 21 | 23 | 20：1 | 11：12 | / |
| Shang et al. | Taiwan | 2020 | Naive | 35（ADHD-CT)/54(ADHD-nonCT)/45(HC) | None | 104.6±11.2/ADHD-nonCT 104.6±13.4/HC-CT 112.7±11.8/HC-nonCT 108.2±12.3 | CT:12±2.6 ；nonCT:11.1J±2.1 | CT：12.4±2.7；nonCT：12.3±3.0 | 96 | HC-CT 49/HC-nonCT 65 | CT：33:6/nonCT:49：8 | CT：36:13/nonCT:38：27 | ADHD-CT C:I:H=12:27:0 ADHD-nonCT C:I:H=18:39:0 |
| Lin et al. | Taiwan | 2019 | Naive | 55/105 | 13 Co-occurring ODD;2 Co-occurring tic disorder;2 Co-occurring adjustment disorder;3 Co-occurring specific phobia | 105.0±13.3/108.6±11.4 | 11.70±2.44 | 12.12±2.95 | 56 | 106 | 47：9 | 68：38 | C:I:H=30:26:0 |
| Icer et al. | Turkey | 2018 | Naive | right | / | <85 | 11.6 ± 2.5 | 13.4 ± 1.76 | 15 | 15 | 12：3 | 10：5 | / |
| Sun et al. | China | 2012 | Naive | right | 5ODD,2CD（conduct disorder）/- | 102.68±10.37/113.52±12.77* | 13.28±1.35 | 13.20±0.95 | 19 | 23 | boy | boy | C:I:H=7:12:0 |
| Cao et al. | China | 2009 | Naive | right | 5ODD,2CD（conduct disorder）/- | 102.7±10.4 /113.5±11.4* | 13.3±1.4 | 13.2±1.0 | 19 | 23 | male | male | 7C:12I |
| Tian et al. | China | 2006 | Eleven of the ADHD patients were medication-free for at least half a year, and one was taken off medication for only 48h | right | / | <80 | 13.91±0.35 | 13.36±0.50 | 8 | 8 | male | male | C:I:H=1:7:0 |
| Han et al. | Korea | 2019 | Naive | / | None | 92.4 ± 12.2/98.4 ± 9.8 | 14.2±1.9（ADHD）/14.6±1.2（ADHD+IGD) | 14.8±2.0 | 55 | 38 | male | male | / |
| Vatansever et al. | UK | 2019 | none | right | None | / | Adult | 20.12±2.28 | / | 172 | / | 59/113 | / |
| Kucyi et al. | USA | 2015 | Refrain from taking them 24 hours prior to testing. Six other subjects had taken psychostimulants in the past and seven were psychostimulant naive | Subjects were right-handed, except for two left-handed ADHD subjects. | / | 119.9±14.0/119.9±11.9 | 24.3±3.9 | 24.2±2.9 | 23 | 23 | 10：13 | 8：15 | C:I:H=12:10:1 |
| McCarthy et al. | Ireland | 2013 | 2naive,10 were drug-free for a mean of 11.6 (SD, 4.2) years but had a history of methylphenidate hydrochloride treatment (mean [SD] months of medication, 20.6 [32.8]). 4were still being treated with methylphenidate and were required to undergo a washout period 48 hours before investigation. | 12/12 | None | 100.9±8.9/104.8±10.9 | 24.5±8.3 | 24.4±8.0 | 16 | 16 | 11：5 | 11：5 | C:I:H=16:0:0 |
| Tan et al. | China | 2019 | Naive | right | Eight patients with the previous history of major depressive disorder and three patients with current diagnosis of general anxiety disorder | 120.72 ± 7.49/121.72 ± 7.92 | 26.5 ± 3.9 | 26.3 ± 3.9 | 69 | 69 | boy | boy | C:I:H=15:54:0 |
| Zhao et al. | China | 2017 | Six participants with ADHD had a history of medicine treatment. Three of the six had stopped the treatment for a long time or had only taken medicine for a short period of time (no <2 months). The other three participants were still being treated and were required to undergo a washout period of 24 h before the MRI scan | right | None | 123.61 ± 9.71/123.13 ± 7.12 | 27.07 ± 5.48 | 25.92 ± 3.77 | 28 | 30 | 15/13 | 17/13 | C:I:H=7:21:0 |
| Ergul et al. | Turkey | 2019 | Being non-medicated for the last 6weeks (8 weeks for fluoxetine) | right | None | / | 26.89 ± 5.94 | 27.24 ± 6.39 | 18 | 21 | 13：5 | 13：8 | / |
| Lin et al. | Taiwan | 2015 | Naive | 22（right)/22 | / | 116.29±14.99/117.71±11.36 | 30.12±9.15 | 30.42±8.95 | 24 | 24 | 11：13 | 11：13 | / |
| Pironti et al. | UK | 2019 | 16 of which were medicated with methylphenidate, while four were not receiving any medication for their ADHD diagnosis. ADHD participants were asked to withhold taking their medication 24 h before and to refrain from consuming alcohol or caffeine containing drinks on the day of the testing. | / | None | 115.26±6.15/119.49±3.27 | 32.2±10.31 | 32.5±5.8 | 20 | 20 | 17：3 | 13：7 | 16C：4I |
| Castellanos et al. | USA | 2008 | Nine patients were currently being treated with stimulants which were discontinued for at least one day prior to scanning. | right | Rule out other AxisI comorbid diagnoses | / | 34.9±9.9 | 31.2±9.0 | 20 | 20 | 16：4 | 14：6 | C:I:H=20:0:0 |

**[Abbreviation](C:/Users/H/AppData/Local/youdao/dict/Application/8.9.6.0/resultui/html/index.html#/javascript:;):** ODD = Oppositional Defiant Disorder; CD = Conduct Disorder; MDD = Major Depressive Disorder; SAD = Separation Anxiety Disorder; SES = Socioeconomic status

**Table S3. Summary of Methods Implemented in Studies Included in Meta-analysis**

| Author | fMRI scanning information | |  |  |  |  | Physiological Regressors | | | Motion Correction | | |
| --- | --- | --- | --- | --- | --- | --- | --- | --- | --- | --- | --- | --- |
|  | machine（tesla) | dur | TR/TE | EO/C | Smooth (mm) | Voxel Size (mm) | global | WM | CSF | despk | 6-parm | other-parm（parameters) |
| Arfuso et al. | 3 | 6min | 2000/30 | O | 6mm | 3× 3× 4 | / | / | / |  |  | 24 parameters |
| Cao et al. | 3 | / | 2000/30 | C | 4mm | 3×3×3 | √ | √ | √ |  | √ |  |
| Castellanos et al. | 3 | 6.5min | 2000/25 | O | 6mm | 3×3×3 | √ | √ | √ |  | √ |  |
| Dias et al. | 3 | 3.5min | 2500/30 | O | / | 3.8×3.8×3.8 | √ | √ | / |  | √ |  |
| Ergul et al. | 3 | 451s | 2000/30 | C | 8mm | 2×2× 4 |  | √ | √ |  | √ |  |
| Han et al. | 3 | 720s | 3000/40 | C | 6mm | 3×3×3 | / | / | / |  | √ |  |
| Hong et al. | 3 | 6min24seconds | 3000/40 | C | 8mm | 1.9×1.9×4 | √ | √ | √ |  | √ | 4 parameters |
| Icer et al. | 1.5 | 9 min 44 s | 2800/25 | C | 6mm | 3×2×2 |  | √ | √ |  |  | √ |
| Jiang et al. | 1.5 | 6min | 2000/40 | C | 6mm | / | / | √ | √ |  | √ |  |
| Karalunas et al. | 3 | 7-10min | 2500/30 | O | 6mm | 3.8×3.8×3.8 | √ | √ | √ |  | √ |  |
| Karalunas et al. | 3 | 7-10min | 2500/30 | O | 6mm | 3.8×3.8×3.8 | √ | √ | √ |  | √ |  |
| Karalunas et al. | 3 | 7-10min | 2500/30 | O | 6mm | 3.8×3.8×3.8 | √ | √ | √ |  | √ |  |
| Kim et al. | 3 | / | 3000/30 | C | 6mm | 3.5 × 3.5 × 3.5 | / | / | / |  |  | 3D motion correction |
| Kucyi et al. | 3 | 10min8s | 3340/30 | O | 6mm | 2.5×2.5×2.5 | √ | √ | √ |  | √ |  |
| Kumar et al. | 3 | / | 2000/30 | / | 8mm | 3.5 × 3.5 × 3.5 |  | √ | √ |  | √ |  |
| Li et al. | 3 | / | 2000/30 | C | 8mm | 3× 3× 3 | √ | √ | √ |  |  | √ |
| Lin et al. | 3 | 6min | 2000/24 | C | 8mm | 4 × 4 × 3 | / | √ | √ |  | √ |  |
| Lin et al. | 3 | 6min | 2000/24 | C | 8mm | 4 × 4 × 3 | / | √ | √ |  |  | 3 parameters |
| Lin et al. | 3 | 6min | 2000/24 | C | 8mm | 4×4×3 | √ | √ | √ |  | / | Friston-24 motion parameters |
| McCarthy et al. | 3 | 7.2min | 2000/28 | / | 8mm | 3× 3× 3 |  | √ | √ |  | √ |  |
| McLeod et al. | 3 | 5min | 2000/30 | O | 6mm | / |  | √ | √ |  | √ |  |
| McLeod et al. | 3 | 5min | 2000/30 | O | 6mm | / |  | √ | √ |  | √ |  |
| Mennes et al. | 3 | 6.5min | 2000/25 | O | 6mm | 3×3×4 | √ | √ | √ |  |  | 3-D motion correction with Fourier interpolation; |
| Mizuno et al. | 3 | 7 minutes 42 s | 2300/30 | C | 6mm | 3.5 × 3× 3 |  | √ | √ |  |  | Friston 24-parameter model |
| Pironti et al. | 3 | 8.75min | 2000/30 | C | 8mm | 3 ×3×3 | √ | √ | √ |  | √ |  |
| Posner et al. | 3 | 2*5min | 2200/30 | C | 6mm | 3.75 × 3.75 × 3.5 |  | √ |  |  | √ | √ |
| Posner et al. | 3 | 2*5min | 2200/30 | C | 6mm | 3.75 × 3.75 × 3.5 | / | / | / |  |  | √ |
| Shang et al. | 3 | 6min | 2000/24 | C | 6mm | 4 ×4 ×3 |  | √ | √ |  |  | √ |
| Sun et al. | 3 | / | 2000/30 | C | 4mm | 3×3×3 mm | √ | √ | √ |  | √ |  |
| Tan et al. | 3 | / | 2000/30 | C | 6mm | 3.2×3.2×3.2 | √ | √ | √ |  |  | Friston's 24 head motion parameters, |
| Tian et al. | 3 |  | 2000/30 | C | 4mm | 3×3×3 |  |  |  |  |  |  |
| Vatansever et al. | 3 | 9min | 3000/minimum full | O | 8mm | 3 ×3 ×3 mm | / | √ | √ |  | √ |  |
| Wang et al. | 1.5 | 6min | 2000/40 | C | 6mm | / | √ | √ | √ |  | √ |  |
| Xie et al. | 1.5 | 6min | 2000/30 | O | / | / | / | √ | √ |  |  | 4 parameters |
| Yang et al. | 1.5 | 6min | 2000/40 | C | 6mm | / | √ | √ | √ |  | √ |  |
| Zhao et al. | 3 | / | 2000/30 | C | 6mm | 3× 3× 3 | √ | √ | √ |  |  | 12 derivative motion parameters |
| Zhou et al. | 3 | 8min | 2000/30 | C | 8mm | 3 × 3×3 |  | √ |  |  | / | 24 parameters |
| Yu et al. | 3 | 8min | 2000/30 | C | 6mm | 3 × 3 × 3 | √ | √ | √ |  | √ |  |

# Appendix 4. Detail of ALE analyses

**Table S4. The detailed information of the studies enrolled for the present ALE analyses**

| **Contrast** | **Number of experiments** | **Number of foci** | **Number of subjects** |
| --- | --- | --- | --- |
| **ADHD>HC** | 28 | 155 | 1479 |
| **ADHD_cortex>HC_cortex** | 18 | 96 | 874 |
| Cortex_older_ADHD>HC | 8 (+) | 44 | 287 |
| Cortex_younger_ADHD>HC | 10 (+) | 52 | 587 |
| **ADHD_subcortex>HC_subcortex** | 10 | 56 | 576 |
| Subcortex_older_ADHD>HC | 2 | 3 | 81 |
| Subcortex_younger_ADHD>HC | 8 | 53 | 495 |
| **ADHD<HC** | 31 (+) | 189 | 2127 |
| **ADHD_cortex<HC_cortex** | 16 (+) | 80 | 904 |
| Cortex_older_ADHD<HC | 6 | 20 | 417 |
| Cortex_younger_ADHD<HC | 10 (+) | 62 | 659 |
| **ADHD_subcortex<HC_subcortex** | 14 | 88 | 1012 |
| Subcortex_older_ADHD<HC | 2 | 7 | 180 |
| Subcortex_younger_ADHD<HC | 12 | 81 | 832 |

***Significance is represented by” (+)”**

Cortex_ADHD > HC = Studies for seeds in the cortex and ADHD>HC);

Cortex_ADHD < HC = Studies for seeds in the cortex and ADHD<HC);

Subcortex_ADHD > HC = Studies for seeds in the subcortex and ADHD>HC);

Subcortex_ADHD < HC = Studies for seeds in the subcortex and ADHD<HC;

Cortex_younger_ADHD > HC = Seeds in the cortex, mean age was younger than 12y, ADHD > HC;

Cortex_older_ADHD > HC = Seeds in the cortex, mean age was greater than or equal to 12y, ADHD > HC;

Cortex_younger_ADHD < HC = Seeds in the cortex, mean age was younger than 12y, ADHD < HC;

Cortex_older_ADHD < HC = Seeds in the cortex, mean age was greater than or equal to 12y, ADHD < HC;

Subcortex_younger_ADHD < HC = Seeds in the subcortex, mean age was younger than 12y, ADHD < HC

Subcortex_younger_ADHD > HC = Seeds in the subcortex, mean age was younger than 12y, ADHD > HC;

Subcortex_older_ADHD < HC = Seeds in the subcortex, mean age was greater than or equal to 12y, ADHD < HC

Subcortex_older_ADHD > HC = Seeds in the subcortex, mean age was greater than or equal to 12y, ADHD > HC;

# Appendix 5. Comparisons of the current results compatible with the previous meta-analysis

**Table S5. Comparisons of including articles**

| Author | Year | Title | Yingxue et.al(21) | Bernis et.al(20) | Samuele et. al(18) | Lingning Liu（35） |
| --- | --- | --- | --- | --- | --- | --- |
| Arfuso et al. | 2019 | Evidence of Altered Habenular Intrinsic Functional Connectivity in Pediatric Adhd. |  |  |  | √ |
| Cao et al. | 2009 | Abnormal Resting-State Functional Connectivity Patterns of the Putamen in Medication-Naive Children with Attention Deficit Hyperactivity Disorder. | √ | √ | √ | √ |
| Castellanoset al. | 2008 | Cingulate-Precuneus Interactions: A New Locus of Dysfunction in Adult Attention-Deficit/Hyperactivity Disorder. |  | √ | √ | √ |
| Diaset al. | 2013 | Reward Circuit Connectivity Relates to Delay Discounting in Children with Attention-Deficit/Hyperactivity Disorder. | √ |  |  | √ |
| Ergulet al. | 2019 | Intrinsic Functional Connectivity in Social Anxiety Disorder with and without Comorbid Attention Deficit Hyperactivity Disorder. |  |  |  | √ |
| Hanet al. | 2019 | Resting-State Fmri Study of Adhd and Internet Gaming Disorder. |  |  |  | √ |
| Hong et al. | 2015 | Functional Dysconnectivity of Corticostriatal Circuitry and Differential Response to Methylphenidate in Youth with Attention-Deficit/Hyperactivity Disorder. | √ | √ | √ | √ |
| Icer et al. | 2018 | Can Functional Connectivity at Resting Brain in Adhd Indicate the Impairments in Sensory-Motor Functions and Face/Emotion Recognition? |  | √ | √ | √ |
| Jiang et al. | 2018 | Characteristics of Functional Connectivity Based on Cerebellum at Resting State on Children with Attention Deficit Hyperactivity Disorder. |  |  |  | √ |
| Karalunas et al. | 2014 | Subtyping Attention-Deficit/Hyperactivity Disorder Using Temperament Dimensions: Toward Biologically Based Nosologic Criteria. | √ |  | √ | √ |
| Kim et al. | 2017 | Balance Deficit and Brain Connectivity in Children with Attention-Deficit/Hyperactivity Disorder. |  |  | √ | √ |
| Kucyi et al. | 2015 | Disrupted Functional Connectivity of Cerebellar Default Network Areas in Attention-Deficit/Hyperactivity Disorder. | √ |  | √ | √ |
| Kumar et al. | 2020 | Neural Network Connectivity in Adhd Children: An Independent Component and Functional Connectivity Analysis of Resting State Fmri Data. |  | √ |  | √ |
| Li et al. | 2014 | Intrinsic Brain Abnormalities in Attention Deficit Hyperactivity Disorder: A Resting-State Functional Mr Imaging Study. | √ | √ | √ | √ |
| Lin et al. | 2015 | Atomoxetine Treatment Strengthens an Anti-Correlated Relationship between Functional Brain Networks in Medication-Na?ve Adults with Attention-Deficit Hyperactivity Disorder: A Randomized Double-Blind Placebo-Controlled Clinical Trial. | √ | √ | √ | √ |
| Lin et al. | 2019 | Increased Functional Segregation Related to the Salience Network in Unaffected Siblings of Youths with Attention-Deficit/Hyperactivity Disorder. |  |  | √ | √ |
| Lin et al. | 2015 | Altered Resting-State Frontoparietal Control Network in Children with Attention-Deficit/Hyperactivity Disorder. | √ | √ | √ | √ |
| McCarthy et al. | 2013 | Attention Network Hypoconnectivity with Default and Affective Network Hyperconnectivity in Adults Diagnosed with Attention-Deficit/Hyperactivity Disorder in Childhood. | √ | √ |  | √ |
| McLeod et al. | 2015 | Functional Connectivity of Neural Motor Networks Is Disrupted in Children with Developmental Coordination Disorder and Attention-Deficit/Hyperactivity Disorder. | √ |  | √ | √ |
| Mennes et al. | 2011 | Resting State Functional Connectivity Correlates of Inhibitory Control in Children with Attention-Deficit/Hyperactivity Disorder. | √ |  | √ | √ |
| Mizuno et al. | 2017 | Catechol-O-Methyltransferase Polymorphism Is Associated with the Cortico-Cerebellar Functional Connectivity of Executive Function in Children with Attention-Deficit/Hyperactivity Disorder. | √ |  | √ | √ |
| Pironti et al. | 2019 | Shared Alterations in Resting-State Brain Connectivity in Adults with Attention-Deficit/Hyperactivity Disorder and Their Unaffected First-Degree Relatives. |  |  |  | √ |
| Posner et al. | 2014 | Dissociable Attentional and Affective Circuits in Medication-Naive Children with Attention-Deficit/Hyperactivity Disorder. | √ | √ |  | √ |
| Posner et al. | 2014 | A Multimodal Mri Study of the Hippocampus in Medication-Naive Children with Adhd: What Connects Adhd and Depression? | √ | √ | √ | √ |
| Shang et al. | 2020 | Effects of the Dopamine Transporter Gene on Striatal Functional Connectivity in Youths with Attention-Deficit/Hyperactivity Disorder. |  | √ |  | √ |
| Sun et al. | 2012 | Abnormal Functional Connectivity between the Anterior Cingulate and the Default Mode Network in Drug-Naive Boys with Attention Deficit Hyperactivity Disorder. | √ | √ |  | √ |
| Tan et al. | 2019 | Alterations of Cerebral Perfusion and Functional Brain Connectivity in Medication-Naive Male Adults with Attention-Deficit/Hyperactivity Disorder. |  |  |  | √ |
| Tian et al. | 2006 | Altered Resting-State Functional Connectivity Patterns of Anterior Cingulate Cortex in Adolescents with Attention Deficit Hyperactivity Disorder. | √ | √ |  | √ |
| Vatansever et al. | 2019 | The Devil Is in the Detail: Exploring the Intrinsic Neural Mechanisms That Link Attention-Deficit/Hyperactivity Disorder Symptomatology to Ongoing Cognition. |  |  |  | √ |
| Wang et al. | 2015 | Abnormal Patterns of Functional Connectivity between Right Temporal Parietal Junction and Whole Brain in Children with Attention Deficit Hyperactivity Disorder. |  |  |  | √ |
| Xie et al. | 2016 | A Study of Rest-Stating Functional Magnetic Resonance Imaging in Children with Attention Deficit Hyperactivity Disorder. |  |  |  | √ |
| Yang et al. | 2013 | Altered Patterns of Functional Connectivity of Posterior Cingulate Cortex on Resting-State Magnetic Resonance Imaging in Children with Attention-Deficit or Hyperactivity Disorder. |  |  | √ | √ |
| Zhao et al. | 2017 | Abnormal Resting-State Functional Connectivity of Insular Subregions and Disrupted Correlation with Working Memory in Adults with Attention Deficit/Hyperactivity Disorder. | √ | √ |  | √ |
| Zhou et al. | 2019 | Abnormal Functional Network Centrality in Drug-Naive Boys with Attention-Deficit/Hyperactivity Disorder. |  |  |  | √ |
| Yu et al. | 2016 | Integrity of Amygdala Subregion-Based Functional Networks and Emotional Lability in Drug-Naïve Boys With ADHD |  | √ | √ | √ |
| Oldehinkel et al. | 2016 | Attention-Deficit/Hyperactivity Disorder Symptoms Coincide with Altered Striatal Connectivity. | DMN,FPN,AN  VAN ,SSN  No sig  adult |  |  | Negtive |
| Mills et al. | 2012 | Altered cortico-striatal-thalamic connectivity in relation to spatial working memory capacity in children with ADHD. | √  Thalamo-striatal disruptions  children |  |  | Data-based |
| [Barber et al.](https://xueshu.baidu.com/s?wd=author:(Barber, Anita D.) &tn=SE_baiduxueshu_c1gjeupa&ie=utf-8&sc_f_para=sc_hilight=person" \o "https://xueshu.baidu.com/s?wd=author:(Barber, Anita D.) &tn=SE_baiduxueshu_c1gjeupa&ie=utf-8&sc_f_para=sc_hilight=person) | 2014 | Connectivity supporting attention in children with attention deficit hyperactivity disorder. |  | √  DMN，ADHD>HC  9 years old |  | Five CON maps and three DMN maps were averaged to  make one mean CON and one mean DMN map. |
| [Costa et al.](https://xueshu.baidu.com/s?wd=author:(Costa Dias, Taciana G.) &tn=SE_baiduxueshu_c1gjeupa&ie=utf-8&sc_f_para=sc_hilight=person" \o "https://xueshu.baidu.com/s?wd=author:(Costa Dias, Taciana G.) &tn=SE_baiduxueshu_c1gjeupa&ie=utf-8&sc_f_para=sc_hilight=person) | 2015 | Characterizing heterogeneity in children with and without ADHD based on reward system connectivity |  | √  AN,both  9 years old |  | Can not match the result with figures |
| Winke et al. | 2015 | The executive control network and symptomatic improvement in attention-deficit/hyperactivity disorder |  | √  CCN,  ADHD>HC  11 years old |  | International Multicenter ADHD Genetics (IMAGE) study |
| [Dardo et al.](https://xueshu.baidu.com/s?wd=author:(Dardo Tomasi) &tn=SE_baiduxueshu_c1gjeupa&ie=utf-8&sc_f_para=sc_hilight=person" \o "https://xueshu.baidu.com/s?wd=author:(Dardo Tomasi) &tn=SE_baiduxueshu_c1gjeupa&ie=utf-8&sc_f_para=sc_hilight=person) | 2012 | Abnormal Functional Connectivity in Children with Attention-Deficit/Hyperactivity Disorder |  | √  AN,ADHD<HC  11 years old |  | ADHD-200 |
| Hoekzema et al. | 2014 | An Independent Components and Functional Connectivity Analysis of Resting State Fmri Data Points to Neural Network Dysregulation in Adult Adhd. | √  FPN no sig  adult |  | √  Use the MNI | Did not survive statistical correction |
| Uytun et al. | 2017 | Default Mode Network Activity and Neuropsychological Profile in Male Children and Adolescents with Attention Deficit Hyperactivity Disorder and Conduct Disorder. | √  DMN,FPN  ADHD>HC  About 12 years old |  |  | Did not survive statistical correction |

#### Table S6. The comparison tables between three meta-analysis

| Author | Year | Search | Inclusion criteria | Exclusion criteria | Analysis | Results |
| --- | --- | --- | --- | --- | --- | --- |
| Yingxue Gao et.al | 2019 | 29 November 2018; PubMed, Web of Science  and EMBASE databases | (1) Had a current ADHD patient group diagnosed according to the DSM-IV, DSM-5, or ICD-10 criteria; (2) had a typically developing (TD) comparison group; (3) directly compared whole-brain seed-based rsFC between ADHD and TD subjects; and (4) reported results as coordinates in stereotactic space. Authors were contacted if the coordinates of seeds or between-group effects were not provided in the study. Moreover, studies with negative findings were also included. | (1) Not seed-based rsFC approach; (2) no whole-brain analyses (restricted to predefined ROIs); (3) coordinates of seed ROIs or between-group effects could not be retrieved; or (4) overlapping samples with the same seeds reported elsewhere. Studies on the same samples but with selection of different seeds were considered separate datasets; studies in which distinct ADHD groups were compared with a single TD group were coded as distinct  datasets. | AES-SDM；  Each seed was then categorized into a seed-network by its location within priori network parcellations | - 25 datasets of 21 studies; - 10 studies for the DMN, 11 studies for the FPN and 8 studies for the AN were included in the meta-analysis; - The results showed that ADHD was characterized by hyperconnectivity between the FPN and regions of the DMN and AN as well as hypoconnectivity between the FPN and regions of the VAN and SSN;   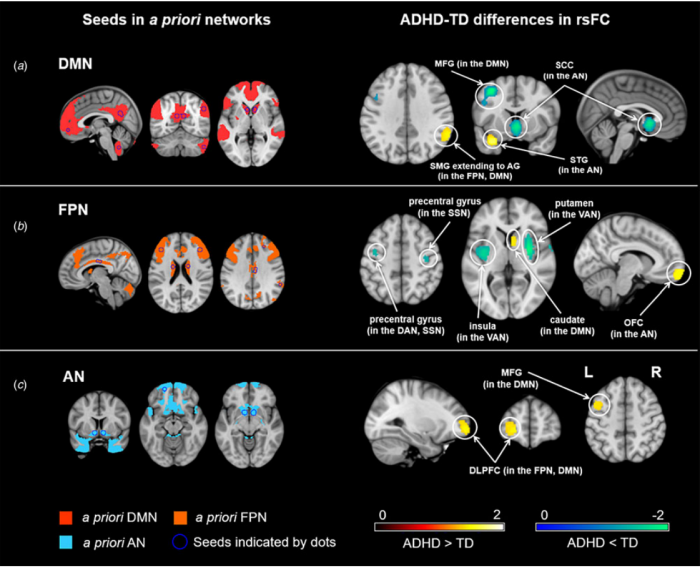 |
| Bernis Sutcubasi et.al | 2020 | 27 January 2020;  PubMed, and Web of Scienc | Original fMRI studies using whole-brain seed-based (seed-seed or seed-voxel) resting-state functional connectivity to compare ADHD and healthy control (HC) groups were eligible for inclusion. | (1) Had no typically developing control group; (2) were based on a non-seed-based method; (3) could not identify standard  resting state networks of interest in the whole sample; and (4) had irretrievable peak RoI coordinates. | Multilevel Kernel  Density Analysis;  Each seed was then categorized into a seed-network by its location within priori network parcellations | - 20 studies met the inclusion criteria, reporting data on 944 separate individuals with ADHD and 1121 healthy controls; - 6 studies for DMN,7 studies for CCN,7 studies for SN, and 9 studies for AN. - Compared to controls, ADHD was associated with disrupted within-default mode network (DMN) connectivity – reduced in the core but elevated in the dorsal medial prefrontal cortex sub-system. Connectivity was elevated between nodes in the cognitive control system. When the analysis was restricted to children and adolescents, additional reduced connectivity was detected between DMN and cognitive control and affective/motivational and salience networks.   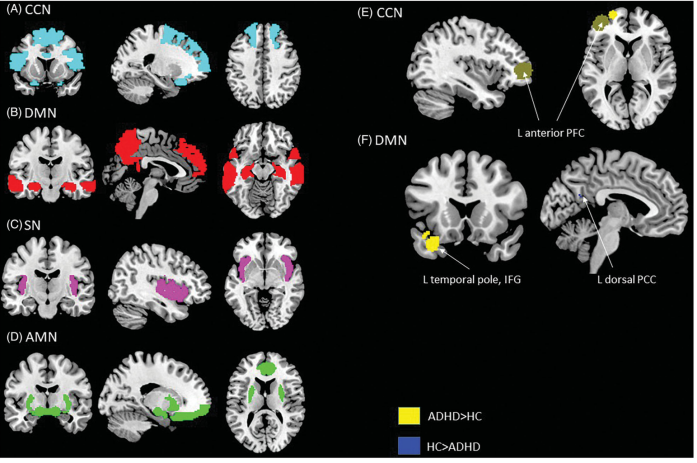  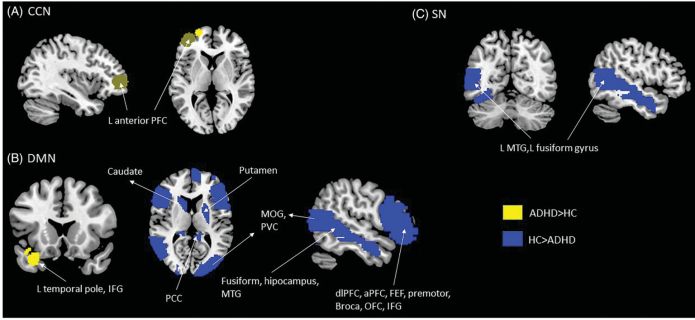 |
| Samuele Cortese  Et.al | 2020 | 9 April 2019;  Pubmed, Ovid MEDLINE®, Biological  Abstracts®, EMBASE Classic+EMBASE, PsycINFO, BIOSIS Previews and Web of Knowledge  (Web of Science (Science Citation Index Expanded), Biological Abstracts, BIOSIS, Food Science  and Technology Abstracts) databases, | Studies using R-fMRI contrasting individuals with ADHD vs.TDC, reporting results as coordinates in standard space, at the whole-brain level. Contacted the corresponding authors of studies retrieved as abstracts/conference proceedings to enquire about their eligibility, and, if needed, gather unpublished information/data necessary for the meta-analysis.  ADHD: Children, adolescents or adults with: 1) a formal categorical diagnosis of ADHD according to the Diagnostic and Statistical Manual of Mental Disorders (DSM III, III-R, IV, IV-TR or 5), or with Hyperkinetic Disorder (HD) as per the International Classification of Diseases (ICD)-10 or previous versions. As per protocol, we deemed eligible studies regardless of the past or current treatment of the participants with ADHD medications.  With no language/type-of-document restrictions | / | ALE;  meta-analyzed studies showing ADHD-related hypo-connectivity and hyper-connectivity in  ADHD, respectively. post-hoc meta-analysis across hypo- and hyper-connectivity, to test convergence in “aberrant” connectivity | - 18 SBC studies,12 non-SBC studies; - No significant spatial convergence of ADHD-related hyper- or hypo-connectivity across studies. This non-significant finding remained after integrating 12 non-SBC studies into the main-analysis and in sensitivity analyses limited to studies including only children or only non-medication naïve patients |
| Ningning Liu et.al | 2022 | October 12th, 2020  Pubmed; Scopus; Web of Science; Embace | (1) had a current formal diagnosis of ADHD according to the DSM-III, DSM-IV, DSM-IV-TR, DSM-5 or ICD-10 criteria; (2) contained a between-group “ADHD versus health control” contrast; and/or (3) correlate with individual level of ADHD symptoms (measured by Adult ADHD Self-Report Scale; Parental Account of Children's Symptoms; Conners' rating scales, and so on). When no peak coordinates of between-group effect were provided, we contacted the authors for detailed information. | (1) not human studies; (2) not ADHD studies; (3) not fMRI studies; (4) not resting-state FC (rsFC) studies; (5) not written in English or Chinese; (6) dataset-based studies. Studies were also excluded if they (1) had no health controls; (2) were not seed-based whole-brain FC studies; (3) did not compare FC of ADHD with healthy control (HC); (4) the same sample and seed regions had been reported in another included study; (5) did not survive statistical correction; (6) had irretrievable peak coordinates when requested. Detail of exclusion studies were listed in the Tabel (Supplementary Appendix 1. Table). | ALE;  Spatial convergence of ADHD-related hyper- and/or hypo- connectivities in cortex and subcortex, across development (<12 and ≥12y). | - In all-age group, only found aberrant activity in “Cortex_ADHD < HC” group. - For the younger, the seeds in cortex consistently showed hyper-connectivity with middle frontal gyrus, whereas hypo-connectivity with left putamen/pallidus/amygdala. - For the older group, more precisely, for adults, the cortex seeds showed a consistent hyper-connectivity with right precuneus/sub-gyral/cingulate gyrus. - The results of independent analyses not only validated the abnormal cortex-cortex and cortex-subcortex FCs pattern in the meta-analysis, but also showed overlaped brain regions with that identified in the meta-analyses. |

# Appendix 6. Supplementary figures

**Figure S1. PRISMA flow chart**


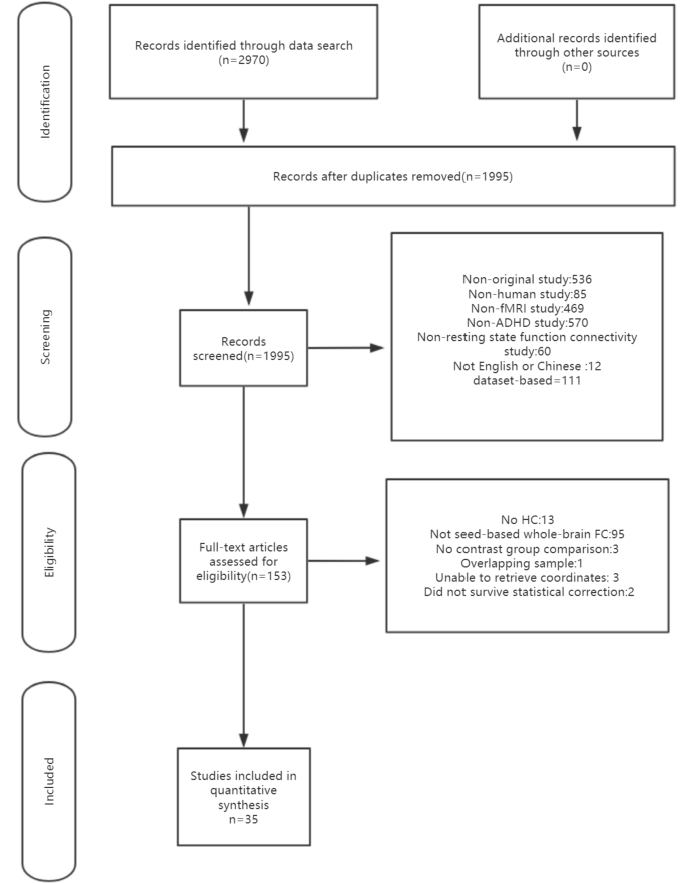


**Figure S2.Anatomical location of foci reported in cortex and subcortex groups**


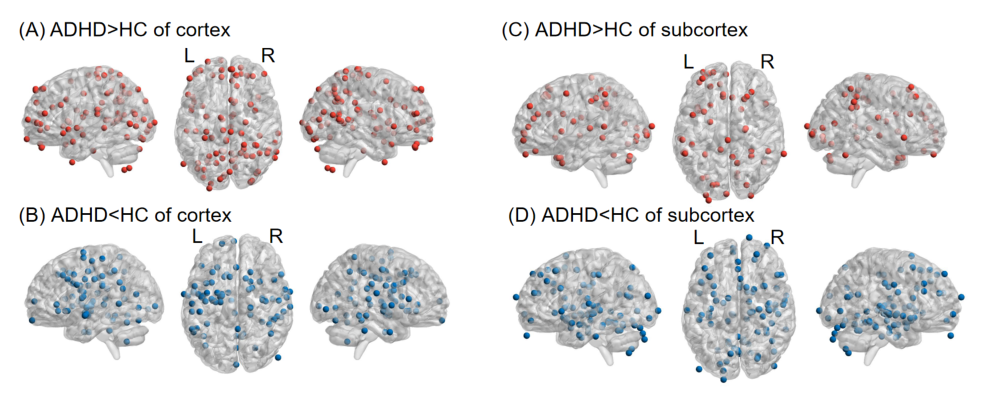


Note: Foci where studies showed hyper-connectivity in attention-deficit/hyperactivity disorderl(ADHD) were shown in red, while foci where studies reported hypo-connectivity in ADHD were illustrated in blue. ADHD = attention-deficit/hyperactivity disorder； HC = Health Control； L = Left; R = Right.

**Figure S3.Anatomical location of foci reported in two age groups**


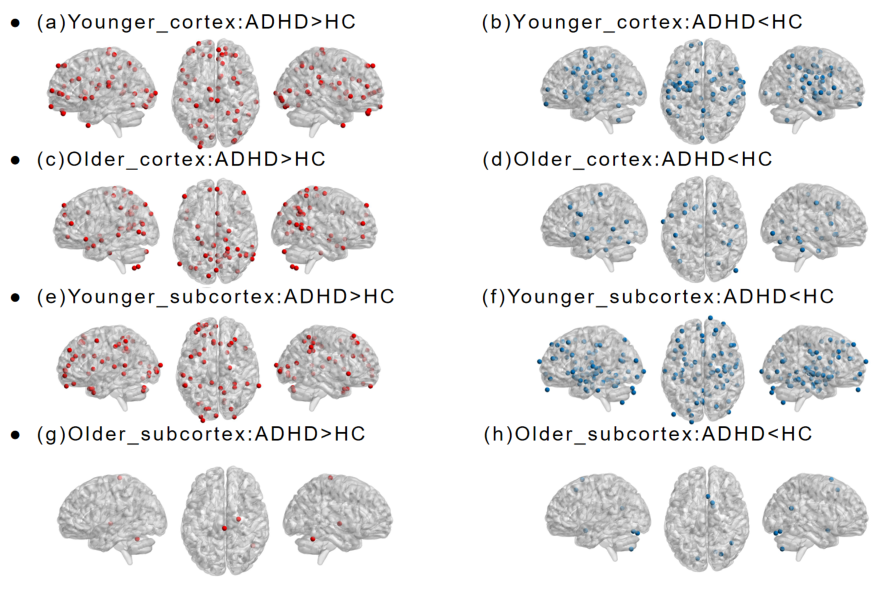


Note: Foci where studies showed hyper-connectivity in attention-deficit/hyperactivity disorderl(ADHD) were shown in red, while foci where studies reported hypo-connectivity in ADHD were illustrated in blue. ADHD = attention-deficit/hyperactivity disorder； HC = Health Control； L = Left; R = Right.

**Figure S4. Abnormal resting-state functional connectivity in ADHD of cortex and subcortex**


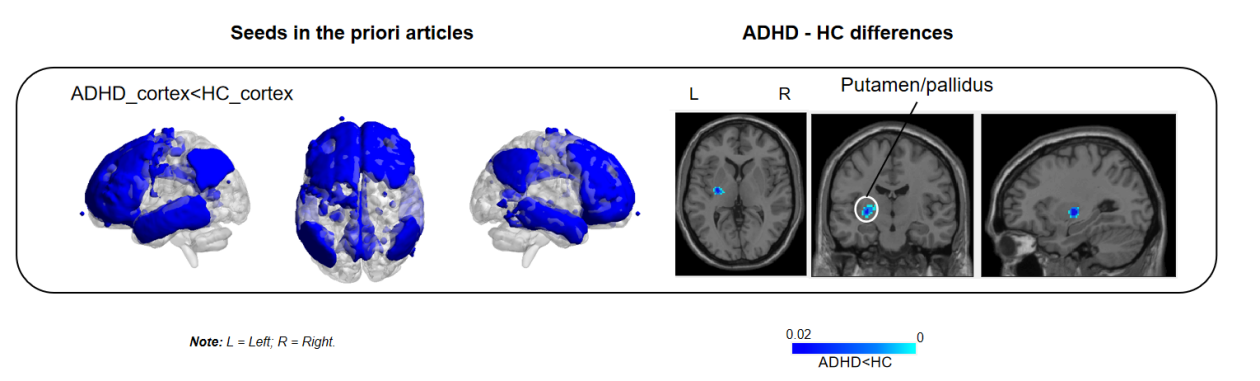


This brain region was located in left putamen/pallidus (1064 mm^3 from (-34,-16,-4) to (-20,-6,8) centered at (-28.9,-11.9,1.8) with 2 peaks，59.4% putamen, 15.8% lateral globus pallidus).

**References:**

[1]. Kaiser, R.H., et al., Large-scale network dysfunction in Major Depressive Disorder: Meta-analysis of resting-state functional connectivity. Other, 2015. 72(6).

[2]. Hoogman, M., et al., Brain Imaging of the Cortex in ADHD: A Coordinated Analysis of Large-Scale Clinical and Population-Based Samples. American Journal of Psychiatry, 2019. 176(7): p. 531-542.

[3]. Dong, H.M., et al., Shifting gradients of macroscale cortical organization mark the transition from childhood to adolescence. Proc Natl Acad Sci U S A, 2021. 118(28).

[4]. Eickhoff, S.B., et al., Coordinate-based ALE meta-analysis of neuroimaging data: A random-effects approach based on empirical estimates of spatial uncertainty. Human Brain Mapping, 2009. 30(9): p. 2907-2926.

[5]. Müller, V.I., et al., Ten simple rules for neuroimaging meta-analysis. Neuroscience & Biobehavioral Reviews, 2017. 84: p. 151-161.

[6]. Null, N., Diagnostic and Statistical Manual of Mental Disorders. Washington, Am Psychiatr Assoc, 1994. 189(1): p. 39-44.

[7]. Yaoxia, G. and C. Taisheng, Chinese Wechsler Intelligence Scale for Children. Chinese Journal of Clinical Psychology, 1994. 002(001): p. 1-6.

[8]. Song, X.W., et al., REST: a toolkit for resting-state functional magnetic resonance imaging data processing. PLoS One, 2011. 6(9): p. e25031.
